# PAK proteins and YAP-1 signalling downstream of integrin beta-1 in myofibroblasts promote liver fibrosis

**DOI:** 10.1038/ncomms12502

**Published:** 2016-08-18

**Authors:** Katherine Martin, James Pritchett, Jessica Llewellyn, Aoibheann F. Mullan, Varinder S. Athwal, Ross Dobie, Emma Harvey, Leo Zeef, Stuart Farrow, Charles Streuli, Neil C. Henderson, Scott L. Friedman, Neil A. Hanley, Karen Piper Hanley

**Affiliations:** 1 Centre for Endocrinology and Diabetes, Institute of Human Development, Faculty of Medical and Human Sciences, University of Manchester, Manchester Academic Health Sciences Centre, Manchester M13 9PT, UK; 2 Research and Innovation Division, Central Manchester University Hospitals NHS Foundation Trust, Manchester M13 9WU, UK; 3 MRC Centre for Inflammation Research, The Queen’s Medical Research Institute, University of Edinburgh, Edinburgh EH16 4TJ, UK; 4 Bioinformatics Core Facility, Faculty of Life Sciences, University of Manchester, Manchester M13 9PL, UK; 5 Respiratory Therapy Area, GlaxoSmithKline, Stevenage SG1 2NY, UK; 6 Wellcome Trust Centre for Cell-Matrix Research, Faculty of Life Sciences, University of Manchester, Manchester M13 9PL, UK; 7 Division of Liver Diseases, Icahn School of Medicine at Mount Sinai, New York, New York 10029, USA

## Abstract

Fibrosis due to extracellular matrix (ECM) secretion from myofibroblasts complicates many chronic liver diseases causing scarring and organ failure. Integrin-dependent interaction with scar ECM promotes pro-fibrotic features. However, the pathological intracellular mechanism in liver myofibroblasts is not completely understood, and further insight could enable therapeutic efforts to reverse fibrosis. Here, we show that integrin beta-1, capable of binding integrin alpha-11, regulates the pro-fibrotic phenotype of myofibroblasts. Integrin beta-1 expression is upregulated in pro-fibrotic myofibroblasts *in vivo* and is required *in vitro* for production of fibrotic ECM components, myofibroblast proliferation, migration and contraction. Serine/threonine-protein kinase proteins, also known as P21-activated kinase (PAK), and the mechanosensitive factor, Yes-associated protein 1 (YAP-1) are core mediators of pro-fibrotic integrin beta-1 signalling, with YAP-1 capable of perpetuating integrin beta-1 expression. Pharmacological inhibition of either pathway *in vivo* attenuates liver fibrosis. PAK protein inhibition, in particular, markedly inactivates the pro-fibrotic myofibroblast phenotype, limits scarring from different hepatic insults and represents a new tractable therapeutic target for treating liver fibrosis.

## Abstract (web summary)

Antifibrotic therapies that target myofibroblast activation are needed to treat chronic liver disease. Here the authors identify an axis of integrin beta-1 expression and Yap-1 and Pak protein signalling that can be interfered with to inhibit myofibroblast function and liver fibrosis *in vivo*.

## Graphical abstract

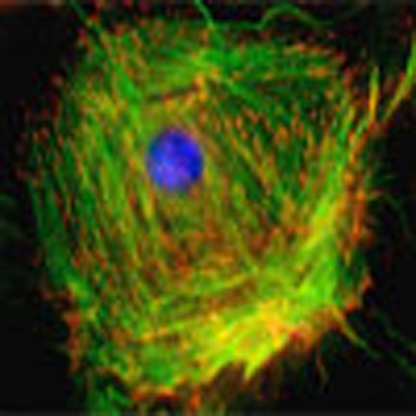

Liver fibrosis is a common step in the majority of chronic liver diseases and is increasing in incidence with a global prevalence of 2% in the general population^1^,^2^. Although potentially reversible during early stages, persistent injury and scar formation results in end-stage cirrhosis, ultimately requiring transplant. In cases in which the underlying cause of hepatic injury cannot be determined and removed, treatment is limited to addressing complications such as portal hypertension, progression to hepatocellular carcinoma and liver failure^3^,^4^,^5^. This problem makes anti-fibrotic liver therapy an urgent priority, but no drugs have been approved to date^6^,^7^.

Hepatic stellate cells (HSCs; liver-specific pericytes) are thought to be a major source of liver myofibroblasts^8^,^9^. In response to injury, quiescent HSCs activate into proliferative, migratory myofibroblasts. Although *in vitro* models of liver fibrosis are an imperfect replica of disease *in vivo*, extraction and culture of HSCs on plastic induces production of collagen-rich extracellular matrix (ECM) characteristic of fibrosis, in part due to the appearance and function of the transcription factor SOX9 (refs 10, 11). It is this pathological ECM that causes contracted scarring and increased organ stiffness.

Although poorly understood, the self-propagating cycle of extracellular stiffness increasing intracellular contractile force is thought to perpetuate fibrotic collagen deposition^12^. Conversely, breaking this relationship could facilitate reversal of liver fibrosis. In other settings, cellular sensing of increased matrix stiffness leads to nuclear activation of the mechanosensitive transcriptional regulator Yes-associated protein 1 (YAP-1)^13^. Interestingly, YAP-1 is expressed in activated HSCs and its inhibition improves scarring in a rodent model of liver fibrosis^14^. Thus, it is important to address directly in liver how ECM might signal from the cell surface to factors in the nucleus, such as YAP-1, as interfering with these pathways might arrest the pro-fibrotic myofibroblast phenotype.

Cells contact the ECM at focal adhesions via cell-surface integrin receptors, heterodimers composed of an alpha and beta subunit^15^. Integrin function is important in HSCs^16^,^17^. In particular, loss of integrin alpha-V in mice or agents that block its function in rats protects against liver fibrosis^18^. However, integrin alpha-V activates the pro-fibrotic cytokine transforming growth factor-β (TGF-β) at the cell surface rather than signalling directly to intracellular mechanosensing pathways^18^. A role for the integrin beta-1 subunit has been proposed but not tested. In skin, TGF-β-dependent mechanisms regulating integrin beta-1 have a major role in fibroblast function during fibrosis and wound healing^19^. Thus, we hypothesize that defining intracellular integrin beta-1 signalling in liver myofibroblasts might identify pathways that mediate mechanotransduction and collagen deposition in fibrosis. Through transcriptomic and experimental analyses of integrin beta-1-deleted and wild-type myofibroblasts, hierarchical clustering identifies mechanosensing pathways involving PAK-1 and YAP-1 as core mediators of pro-fibrotic integrin signalling. Pharmacological inhibition of either pathway lessens the pro-fibrotic myofibroblast phenotype *in vitro* and attenuates liver fibrosis *in vivo*. These data implicate PAK protein inhibitors as particularly tractable for repositioning from clinical trials in cancer as new anti-fibrotic agents, avoiding the need to target integrin-ECM interactions, which are ubiquitous throughout healthy and diseased tissue.

## Results

### Integrin beta-1 is required for pro-fibrotic HSCs

**Figure 1:**
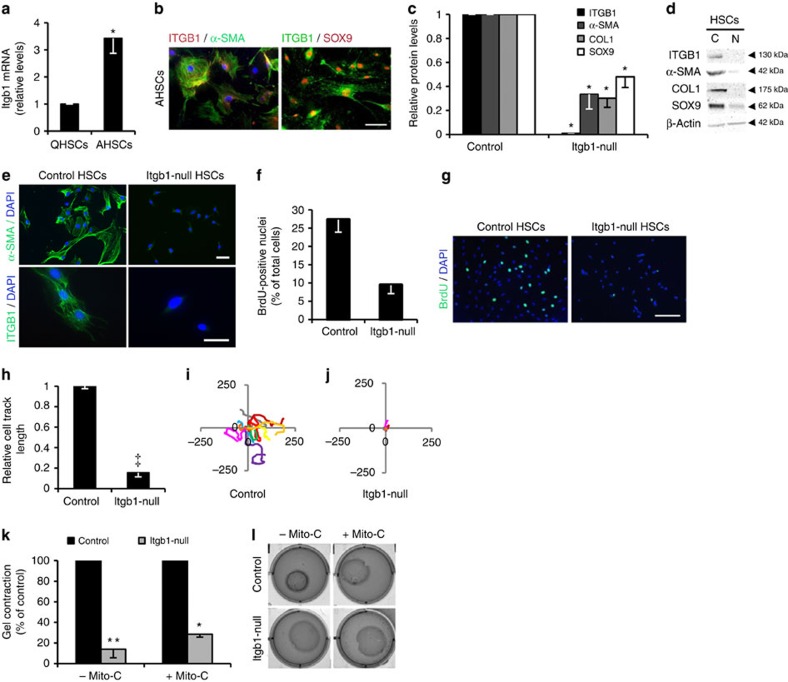
Integrin beta-1 is increased in activated HSCs and required for phenotype and function. (**a**) integrin beta-1 is increased in activated (A) compared with quiescent (Q) rat HSCs by qRT–PCR. (**b**) Immunofluorescence of rat AHSCs shows integrin beta-1 costained in cells with pro-fibrotic markers α-SMA (green; DAPI as blue nuclear counterstain) and SOX9 (red). (**c**,**d**) Activated mouse HSCs (‘Control’) show decreased protein levels for α-SMA, COL1 and SOX9 by immunoblotting following the loss of integrin beta-1 (‘*Itgb1*-null’). Quantification from *n*≥3 experiments in **c** and example immunoblots shown in **d**. (**e**) α-SMA is lost by immunofluorescence from activated mouse HSCs (‘Control’) following integrin beta-1 inactivation (‘*Itgb1*-null’). DAPI (blue) used as nuclear counterstain. (**f**,**g**) Proliferation measured by BrdU incorporation of activated mouse HSCs (‘Control’) declines following integrin beta-1 inactivation (‘*Itgb1*-null’). Quantification from *n*≥3 experiments in **f** with example immunofluorescence in **g**. DAPI, blue nuclear counterstain. (**h**,**j**) Migration of activated mouse HSCs (‘Control’) over 24 h is almost entirely attenuated following the loss of integrin beta-1 (‘*Itgb1*-null’). Quantification from *n*=3 biological replicates with 30–77 cells in each experiment in **h** with an individual example of migratory tracks (in μm) shown in **i**,**j**. (**k**,**l**) Contractile properties of activated mouse HSCs (‘Control’) are markedly attenuated after inactivation of integrin beta-1 (‘*Itgb1*-null’). Quantification of gel contraction from *n*=3 experiments is shown in **k** in the presence or absence of mitomycin-C (Mito-C) and example images shown in **l**. Scale bars, 50 μm. Two-tailed unpaired *t*-test was used for statistical analysis. Data are shown as means±s.e.m. *P<0.05, **P<0.01, ^‡^*P*<0.001. C, control-activated HSCs; N, integrin beta-1-null HSCs.

HSCs extracted from the liver and cultured on plastic for 7–10 days model myofibroblast activation with the appearance of α-Smooth muscle actin (α-SMA), Sex determining region Y-box 9 (SOX9) and the major fibrotic ECM protein, Collagen type 1 (COL1) (refs 10, 11, 20). *Itgb1* increased in parallel with these markers (Fig. 1a,b and Supplementary Fig. 1a,b). Interestingly, while integrin alpha-V was increased consistent with previous findings^18^, ITGA11 was more strikingly enhanced following activation of myofibroblasts when it co-precipitated with integrin beta-1 (Supplementary Fig. 1c–e). To probe the consequences of this integrin signalling, we isolated HSCs from *Itgb1*^fl/fl^; CreER^TM^ mice and treated the cells with tamoxifen to inactivate *Itgb1* or dimethyl sulfoxide (DMSO) as control^21^. Following culture on plastic, activated HSCs lacking integrin beta-1 had a 50–70% reduction in fibrotic markers α-SMA, COL1 and SOX9 (Fig. 1c–e). Stress fibres (marked by F-actin) were greatly reduced and the myofibroblast-like appearance was lost in the mutant HSCs which had a rounded phenotype and were less than half as proliferative as control myofibroblasts (Fig. 1f–g and Supplementary Fig. 1f). Loss of integrin beta-1 did not affect cell viability or, in contrast to previous data^22^, apoptosis (Supplementary Fig. 1g,h). Over 24 h single tracked integrin beta-1-deficient myofibroblasts were markedly less migratory than control cells (Fig. 1h,j). We also examined the consequences of inactivating integrin signalling in myofibroblasts that had already developed a pro-fibrotic phenotype; *Itga11* knockdown or later recombination of the *Itgb1* allele diminished SOX9 and COL1 levels (Supplementary Fig. 2a–c), and also drastically reduced HSC migration (Supplementary Fig. 2d–f). Loss of integrin beta-1 signalling in prior activated HSCs also disabled collagen gel contraction independent of cell proliferation (Fig. 1k,l). Collectively, these data demonstrate that abrogation of integrin beta-1 signalling, potentially as ITGA11B1 heterodimers, both blocks and reverses major pro-fibrotic features of liver myofibroblasts *in vitro*, including key markers, cell migration, proliferation, contractility and collagen production.

### Downstream pathways of integrin beta-1 in activated HSCs

**Figure 2:**
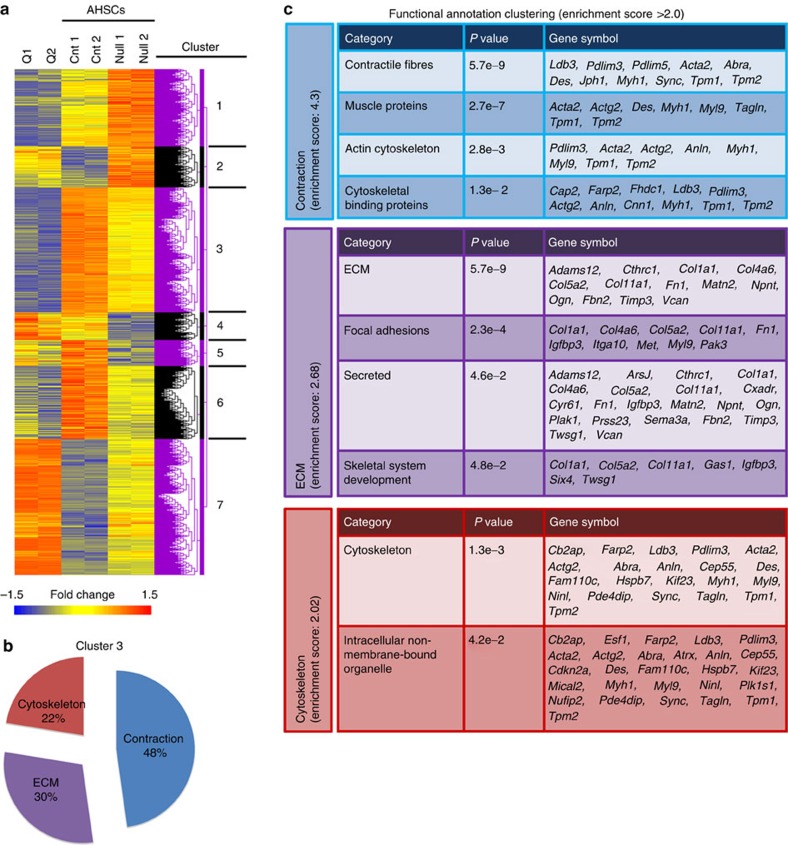
Integrin beta-1 is required for multiple pro-fibrotic features in activated HSCs. (**a**) Heatmap and cluster analysis showing gene expression changes (1.5-fold; *P*<0.05) in biological duplicates of activated (A) control (‘Cnt’) HSCs and following integrin beta-1 inactivation (‘Null’). Quiescent (Q) HSCs are also included for comparison. Seven clusters were identified based on upregulated (red), downregulated (blue) and intermediate (yellow) gene expression. (**b**,**c**) Functional annotation by gene ontology for enrichment in Cluster 3. Proportions are shown in **b**. Individual categories and the genes underlying them are shown in **c**.

These findings made it logical to explore the intracellular events of integrin beta-1 signalling in hepatic myofibroblasts. We analysed the transcriptome of integrin beta-1-deleted and wild-type myofibroblasts, cultured on plastic for 7 days, versus their freshly isolated quiescent HSC counterparts. Following hierarchical analysis of replicate samples, displayed by heatmap, differentially expressed genes clustered into seven categories (Fig. 2a and microarray data set E-MTAB-4810). Loss of integrin beta-1 did not convert activated myofibroblasts back to a quiescent phenotype, but a largely intermediate state consistent with recent data on a separate population of inactivated HSCs that arise during the resolution of murine liver fibrosis^4^. The two largest clusters contained: 225 genes upregulated in activated myofibroblasts whose expression was diminished without integrin beta-1 signalling (Cluster 3); and the converse, 245 genes downregulated in activated myofibroblasts whose expression was partially restored without integrin beta-1 signalling (Cluster 7). Consistent with our phenotypic and functional data, Cluster 3 overrepresented genes involved in organizing contractility, ECM signalling and the cytoskeleton (Fig. 2b,c and Supplementary Fig. 3) whereas Cluster 7 genes included categorization as inflammation, cell proliferation, adhesion and migration, and wounding (Supplementary Fig. 4). Cluster 2 contained 73 genes which became upregulated in inactivated myofibroblasts to levels beyond those in quiescent HSCs and was also annotated by wound healing and the regulation of cell proliferation (Supplementary Fig. 5).

We reasoned that further analysis of Cluster 3 might prioritize mechanosensing pathways which could be inhibited beneficially to reduce collagen deposition in liver fibrosis. In the top 20 gene-ontology terms most significantly associated with Cluster 3 (Supplementary Fig. 6), Group I PAKs, for which there are three family members, and myosin light chain 9 (MYL9), the contractility protein, stood out as annotated 13 times. Interestingly, during cell transformation PAK proteins can signal via the mechanosensitive transcriptional coactivator, YAP-1 (ref. 23); while in cancer-associated fibroblasts, YAP-1 can mediate the effect of matrix stiffness on enhancing MYL9 production^24^. Although PAK proteins and YAP-1 have been detected in HSCs^25^,^26^, PAK, YAP-1 and MYL9 have not previously been reported in integrin beta-1 signalling or collectively as regulators of HSC function/liver fibrosis and therefore we set about investigating these factors in greater detail.

### Myocontractile HSCs require integrin beta-1 and YAP-1

**Figure 3:**
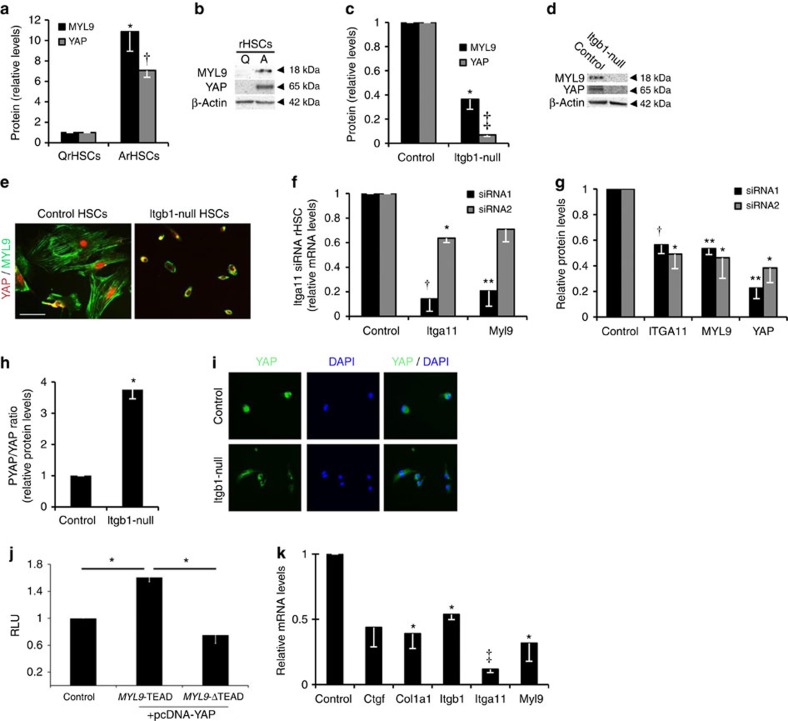
YAP-1 and MYL9 mediate pro-fibrotic aspects of liver fibrosis in activated HSCs. (**a,b**) Quantified increase in total MYL9 and YAP-1 protein levels on activation of rat HSCs from *n*≥3 experiments (**a**) with representative immunoblot shown in **b**. (**c–e**) Total YAP-1 and MYL9 are diminished in activated mouse HSCs (‘Control’) following integrin beta-1 loss (‘*Itgb1*-null’). Quantification from *n*=3 experiments shown in **c** with representative immunoblot in **d**. In the immunofluorescence in **e**, note the rounded inactivated appearance of the *Itgb1*-null cells. The remaining total YAP-1 signal is more cytoplasmic (see **h**,**i**). Scale bar, 50 μm. (**f**,**g**) *Itga11* knockdown in activated rat HSCs using two different siRNA oligos. Data for each oligo are shown relative to its own scrambled control in either black or grey (*n*=3 for each). (**f**) Detection of *Myl9* transcripts was diminished to an almost identical extent as for *Itga11*. (**g**) Protein detection of MYL9 and total YAP-1 was also diminished following *Itga11* knockdown. (**h**,**i**) The proportion of phosphorylated YAP (PYAP, inactive form), is increased following integrin beta-1 loss (‘*Itgb1*-null’) from activated mouse HSCs (‘Control’; *n*=3 experiments) and localises more predominantly to the cytoplasm (**i**). DAPI, blue nuclear counterstain, is shown. Scale bar, 50 μm. (**j**) Luciferase activity (in relative light units; RLU) following co-transfection of constructs containing the wild-type (*MYL9-*TEAD) or mutated (*MYL9-*ΔTEAD) TEAD motif from the 3′-untranslated region of the *MYL9* gene with empty vector (Control) or YAP expression vector. Results are normalized to a *Renilla* vector and expressed relative to the control MYL9 luciferase construct without YAP. (**k**) Transcript levels by qRT–PCR following inhibition of YAP-TEAD interaction using VP in activated rat HSCs expressed relative to DMSO control. Two-tailed unpaired *t*-test was used for statistical analysis. Data are shown as means±s.e.m. *P<0.05, **P<0.01, ^†^*P*<0.005, ^‡^*P*<0.001.

**Figure 4:**
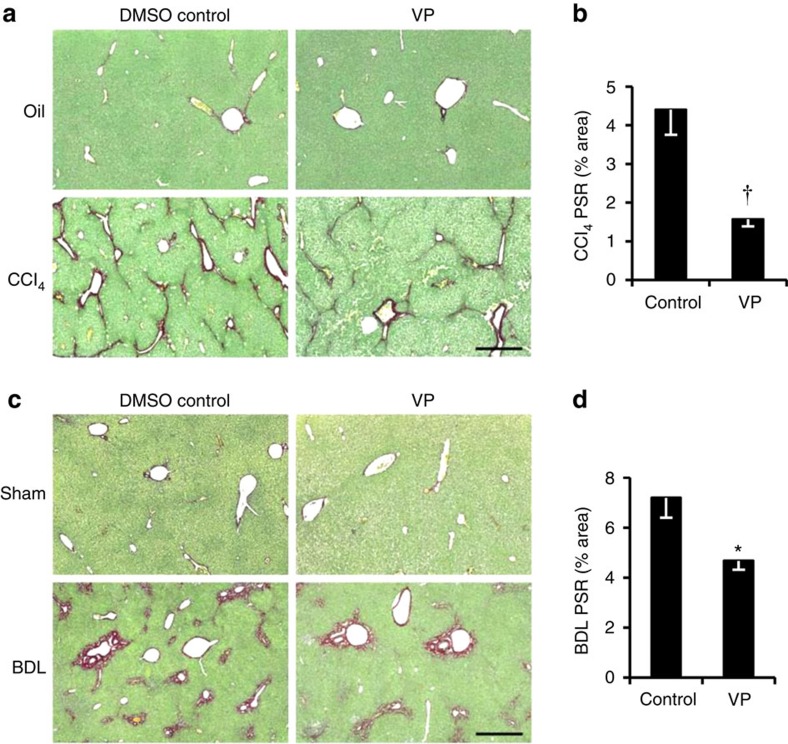
Pharmacological inhibition of YAP-1 with VP improves liver fibrosis *in vivo*. (**a**) PSR staining (collagen deposition in red) counterstained with fast green in olive oil control (Oil; top) or chronic CCl_4_-induced fibrosis (bottom) in mice treated with DMSO (*n*=4) or VP (VP; *n*=3). (**b**) Quantification of surface area covered by the PSR staining in **a**. (**c**) PSR staining (collagen deposition in red) in sham-operated mice (Sham; top) or BDL to induce peribiliary fibrosis (bottom) with control DMSO (*n*=7) or VP (*n*=5) treatment. (**d**) Quantification of surface area covered by the PSR staining in **c**. Scale bar, 500 μm. Liver weight was unaffected in both models treated with VP. Two-tailed unpaired *t*-test was used for statistical analysis. Data are shown as means±s.e.m. *P<0.05, ^†^*P*<0.005.

YAP-1 and MYL9 were markedly increased following activation of wild-type, but not integrin beta-1-deficient liver myofibroblasts (Fig. 3a–e). Similarly, MYL9 and YAP-1 transcripts and protein were diminished following abrogation of *Itga11* (Fig. 3f,g and Supplementary Fig. 8). Phosphorylation of YAP-1 blocks its function by promoting nuclear export. Lack of integrin beta-1 caused nearly fourfold increase in phosphoYAP-1 and greater cytoplasmic localization of total YAP-1 (Fig. 3h,i). YAP-1 functions as a coactivator of the TEA domain family of transcription factors (TEADs)^27^. We identified a conserved TEAD consensus motif in the 3′-untranslated region of *MYL9* that was bound by a complex that included YAP-1 and was required for YAP-1 induction of a *MYL9* reporter construct (Fig. 3j and Supplementary Fig. 7). Verteporfin (VP) inhibits YAP-1 function by blocking its interaction with TEAD^27^. Treating activated myofibroblasts with VP reduced expression of *Col1A1*, *Myl9* and *Ctgf*, a YAP-1 target gene in fibroblasts and cancer cell lines^28^, and an indicator of HSC activation^29^,^30^ (Fig. 3k). Interestingly, functional YAP was also required for full *Itga11* and *Itgb1* expression implying an auto-regulatory loop to perpetuate the pro-fibrotic myofibroblast phenotype. Collectively, these data indicate that integrin beta-1, potentially in partnership with ITGA11, signals via YAP-TEAD to regulate the myocontractility factor, MYL9, in activated hepatic myofibroblasts. To explore whether blocking YAP-1 function might ameliorate liver fibrosis, we administered VP to mice in two different models of liver fibrosis. To model parenchymal hepatic injury we induced fibrosis for 6 weeks using carbon tetrachloride (CCl_4_) injections while treating with VP during the last 3 weeks. To model peribiliary sclerosing disorders, fibrosis was induced for 2 weeks using bile duct ligation (BDL) with VP treatment during the last week. In neither model was VP toxic to hepatocytes as assessed by serum alanine aminotransferase (ALT) levels in control animals (Supplementary Figs 9a and 10a). However, while VP restored ALT and bilirubin to background levels only in the BDL model (Supplementary Fig. 10a), fibrotic collagen deposition, assessed by picro sirius red (PSR) staining, was significantly reduced in both injury settings (Fig. 4a–d) consistent with trends post-treatment in hydroxyproline content (Supplementary Figs 9b and 10b). While VP did not alter myofibroblast numbers or inflammation in the CCl_4_ model of parenchymal liver damage, both showed trends to amelioration post-BDL (Supplementary Fig. 10e–h).

### PAK proteins contribute to pro-fibrotic myofibroblasts

**Figure 5:**
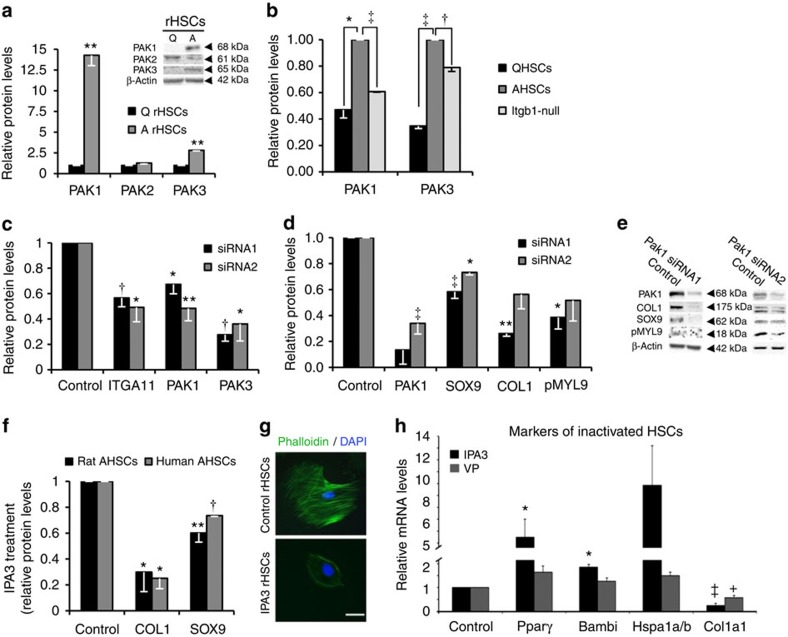
Integrin beta-1 inactivation identifies PAK signalling as a requirement for liver fibrosis. (**a**–**f**) Quantification of immunoblots showing: (**a**) increased protein levels of PAK-1 and PAK-3 on activation of rat HSCs compared with quiescent (Q) cells; (**b**) decreases in both PAK-1 and PAK-3 following the loss of integrin beta-1 (‘*Itgb1*-null’) in activated mouse HSCs (AHSCs). Levels in mouse QHSCs are shown for comparison. (**c**) Expression of PAK-1 and PAK-3 proteins are similarly diminished following Itga11 knockdown using two independent siRNAs in activated mouse HSCs relative to control (scrambled siRNA). (**d**,**e**) Decreases in the levels of activated HSC markers, SOX9, COL1 and phosphoMyl9 (PMYL9) following PAK-1 abrogation by siRNA1 in activated rat HSCs relative to their respective scrambled control levels (‘Control’). Similar decreases were observed with a second independent siRNA (siRNA2). Representative immunoblot is shown in **e**. (**f**) Decreases in COL1 and SOX9 following the inhibition of group I PAKs using IPA3 treatment in rat and human-activated HSCs compared with DMSO control. (**g**) IPA3 treatment of activated rat HSCs disrupts the actin cytoskeleton (phalloidin staining in green). Note, the rounded cell appearance following IPA3 treatment similar to Fig. 3e and Supplementary Fig. 1f. Scale bar, 50 μm. (**h**) Relative expression levels by qRT–PCR following VP or IPA3 treatment of activate rat HSCs for three genes identified as markers of HSC inactivation^4^. As expected, Col1a1 levels were decreased in response to both treatments. For experiments in **a**–**h**, *n*=3 or 4. Two-tailed unpaired *t*-test was used for statistical analysis. Data are shown as means±s.e.m. *P<0.05, **P≤0.01, ^†^*P*<0.005, ^‡^*P*≤0.001.

There are three group I PAK members (PAK-1, PAK-2 and PAK-3). In contrast to PAK-2, PAK-1 and PAK-3 were both increased on myofibroblast activation (Fig. 5a), however, this induction was significantly reduced in the absence of integrin beta-1 (Fig. 5b) and following abrogation of *Itga11* (Fig. 5c and Supplementary Fig. 8). Abrogating *Pak1* or *Pak3* using siRNAs in myofibroblasts with intact integrin signalling reduced SOX9 and COL1 (Fig. 5d–e and Supplementary Fig. 11). PAK can phosphorylate MYL proteins^31^. Although we could not detect any alterations in total MYL9 levels, its activated phosphorylated form, phosphoMYL9, was also significantly decreased when *Pak1* was knocked down (Fig. 5d,e). The catalytic activity of PAK-1 and PAK-3 can be inhibited allosterically by 1,1’-dithiodi-2-naphthol (IPA3) (ref. 32). Treating activated rat and human hepatic myofibroblasts with IPA3 significantly reduced COL1 and SOX9 levels and blocked actin filament formation resulting in a rounded cell phenotype very similar to *Itgb1*-null HSCs (Fig. 5f,g and Supplementary Fig. 1f). IPA3 treatment did not affect cell viability or further reduce fibrotic markers in *Itgb1*-null activated HSCs, implying the effects of IPA3 were mediated by the same pathway as integrin beta-1 (Supplementary Fig. 12). In contrast to VP treatment, IPA3 also increased expression of several markers of inactivated HSCs^4^,^5^, while both VP and IPA3 reduced *Col1a1* expression (Fig. 5h).

### Inhibition of PAK-1 improves liver fibrosis *in vivo*

**Figure 6:**
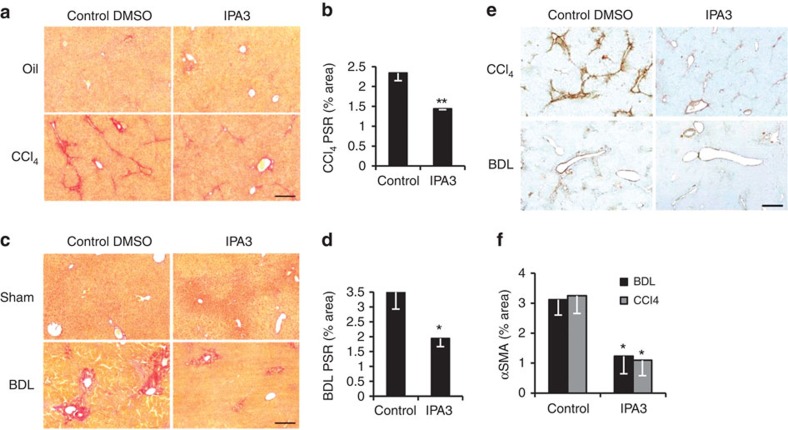
Pharmacological inhibition of PAK-1 improves liver fibrosis in rodents. (**a**) PSR staining (collagen deposition in red) in olive oil control (Oil; top) or chronic CCl_4_-induced fibrosis (bottom) with DMSO (*n*=5) or IPA3 (*n*=4) treatment. (**b**) Quantification of surface area covered by the PSR staining in **a**. (**c**) PSR staining (collagen deposition in red) in sham-operated mice (Sham; top) or BDL to induce peribiliary fibrosis (bottom) with control DMSO (*n*=6) or IPA3 (*n*=7) treatment. (**d**) Quantification of surface area covered by the PSR staining in **c**. (**e**) Immunohistochemistry for α-SMA (brown; activated HSC/myofibroblast marker) in mouse livers following CCl_4_ (top) or BDL (bottom) induced fibrosis. (**f**) Quantification of surface area covered by α-SMA staining was reduced following IPA3 treatment in both models (*n*=5 for all animal groups). Scale bar, 500 μm. Two-tailed unpaired *t*-test was used for statistical analysis. Data are shown as means±s.e.m. *P<0.05, **P≤0.01.

To investigate whether blocking PAK-1 function might improve liver fibrosis, we administered IPA3 intraperitoneally to mice during the last four weeks of an 8-week regime of CCl_4_ injections. IPA3 significantly reduced fibrotic collagen deposition in the hepatic parenchyma by PSR staining while liver weight was unaffected (Fig. 6a,b and Supplementary Fig. 13a). Liver hydroxyproline content was also markedly reduced (Supplementary Fig. 13e). In addition, in BDL-induced liver fibrosis the marked peribiliary collagen deposition in control animals was greatly improved in those animals that had been treated with three injections of IPA3 during the second week (Fig. 6c,d). Liver weight was unaltered (Supplementary Fig. 13b). In both models, IPA3 also reduced the amount of α-SMA staining in the liver implying reduced myofibroblast activation (Fig. 6e,f) consistent with the increased expression of markers for HSC inactivation^4^,^5^ (Fig. 5h). Although no clear significant improvements in liver function were detected in serum (assessed by ALT and bilirubin levels), IPA3 treatment showed no evidence of liver toxicity.

**Figure 7:**
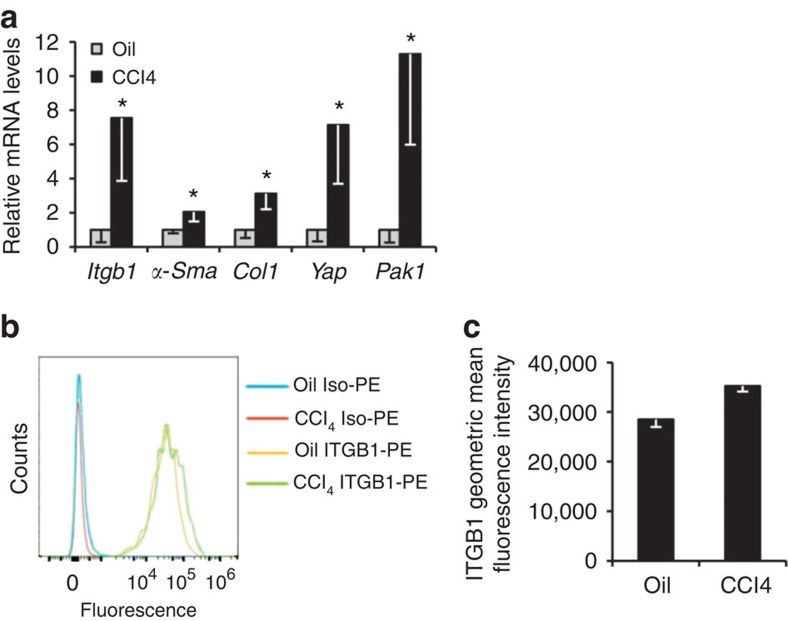
Analysis of *in vivo* activated HSCs. (**a**) Expression analysis by qRT–PCR of *in vivo* activated HSCs extracted from wild-type mice following CCl_4_ injections compared with olive oil control. (**b**,**c**) FACS analysis for integrin beta-1 on *in vivo* activated HSCs extracted from Pdgfrb-BAC-eGFP mice following CCl_4_-induced fibrosis compared with olive oil control. Fluorescence intensity is shown using a phycoerythrin (PE)-conjugated antibody to integrin beta-1 (integrin beta-1-PE) and isotype control (Iso-PE) (**b**). Graphical representation of integrin beta-1 fluorescence demonstrating increased integrin beta-1 on unpermeabilized live HSCs the context of liver fibrosis (**c**). All experiments are *n*=4. Two-tailed unpaired *t*-test was used for statistical analysis. Data in bar charts show means±s.e.m. *P<0.05, **P<0.01.

Taken together, these data demonstrate integrin beta-1 upstream of YAP-1 and PAK protein signalling in HSCs *in vitro*; the importance of all three to the pro-fibrotic phenotype of HSCs *in vitro*; and that inhibiting YAP-1 and PAK protein function *in vivo* ameliorates liver fibrosis. As a final corroborative step we examined HSCs isolated from liver fibrosis *in vivo*. We followed an established protocol^33^,^34^ for extracting and analysing HSCs following a total of four intraperitoneal injections of CCl_4_ or olive oil control every 2 days. Consistent with all the preceding data, *Itgb1*, *α-Sma*, *Col1*, *Yap1* and *Pak1* were all increased in HSCs from the fibrotic liver (Fig. 7a). Moreover, in the livers of platelet-derived growth factor receptor beta (Pdgfrb) knock-in reporter mice (Pdgfrb-BAC-eGFP) (ref. 18) there was a greater detection of integrin beta-1 on the surface of live HSCs following 6-week CCl_4_ induction of liver fibrosis (Fig. 7b,c).

## Discussion

Liver fibrosis ultimately ending in cirrhosis complicates many chronic liver diseases and urgently needs new therapeutic entities. Although integrin beta-1 has previously been intimated as important in HSC function as a putative partner for integrin alpha-V, difficulty with myofibroblast-specific integrin beta-1 inactivation still precludes its direct investigation in liver fibrosis *in vivo* as described by others^18^. Consequently, in this study we have provided *in vitro* evidence for ITGA11-integrin beta-1 heterodimers in liver myofibroblasts, where loss of either heterodimer led to an inactivated HSC phenotype remarkably similar to recent data on fibrosis resolution^4^,^5^. Compared with genetic inactivation of integrin beta-1, reliance on *in vitro* transient knockdown prevented study of ITGA11 in cell contraction or migration. Future genetic inactivation of ITGA11 will facilitate these investigations along with *in vivo* analyses. Our data also do not preclude important interactions between integrin beta-1 and other ITGA subunits. Although *Itga3* was increased in activated myofibroblasts we could not detect an interaction with integrin beta-1. Although relatively little is known about the molecular mechanisms by which ITGA11B1 functions, studies in fibroblasts from other lineages support our data by invoking potential regulation via mechanical stress^35^,^36^.

Targeting integrin beta-1 allowed us to identify downstream intracellular pathways involving YAP-1 and PAK proteins. In the liver, YAP-1 has been identified as an important regulator of organ size and regeneration^37^. More recently, studies have indicated YAP-1 is required for HSC activation *in vitro*, whereas *in vivo*, a single dose of the YAP-1 inhibitor VP improved scarring in mice with 4 week CCl_4_-induced fibrosis^14^. Although our data from both CCl_4_ and BDL models of fibrosis are in broad agreement with this latter study, in our hands the short acting nature of VP required multiple weekly doses to improve fibrosis in rodents; a single injection had no effect.

Molecularly, while there was marked overlap, we did not find evidence for a hierarchical relationship between PAK proteins and YAP-1. Notably, YAP-1 signalling preserved expression of both integrin beta-1 and ITGA11 implying auto-regulation similarly to YAP-1’s function in cancer-associated fibroblasts, where MYL9 was a key regulator of the ECM and tumour invasion^24^. Interestingly, PAK-1 and PAK-2 appear to play opposing roles in models of breast cancer. Inactivation of PAK-2 enhanced phosphorylation of MYL and cell invasiveness, whereas PAK-1 inactivation had the opposite effect^38^. These data are reminiscent of our own observations of quiescent (PAK-2^+^/PAK-1^−^ and MYL9^−^) versus activated HSCs (PAK-2^−^/PAK-1^+^ and MYL9^+^). In keeping with these opposing roles, studies have indicated PAK-2 negatively regulates PAK-1 activity^39^. Because of a tendency for mice to spontaneous recovery following CCl_4_ injections, it is difficult to decipher agents that reverse liver fibrosis rather than attenuate or inhibit its development. The *in vitro* data here all support reversal of established pro-fibrotic myofibroblasts to a more inactivated state. While additional signalling pathways might also influence PAK-1 and YAP-1 in HSCs, *in vivo*, integrin beta-1 and PAK-1 (and YAP-1) were all co-increased in HSCs from fibrotic livers and PAK protein inhibition clearly lessened fibrosis in two different models of liver injury. While IPA3 is unsuitable for clinical development, other Group I PAK inhibitors are already in clinical trials for various cancers^40^. Moreover, drugs in current clinical use exhibit PAK inhibitory activity and may be suitable for therapeutic repositioning^41^,^42^. In summary, these data report new mechanisms for liver fibrosis involving the group 1 PAK proteins and YAP-1 as important effectors of integrin-dependent signalling in hepatic myofibroblasts and highlight PAK inhibitors as potential new agents with which to target liver fibrosis in patients.

## Methods

### Animals

The *Itgb1*^fl/fl^ and CreER^TM^ mice have been described previously^21^. Adult (8 weeks old) male mice were used and genotyping was performed from DNA prepared from ear clips using the REDExtract-N-Amp Tissue PCR Kit (Sigma) and PCR primers for integrin beta-1 forward 5′-TTCTGCAAGTGTGGTG-3′ and reverse 5′-TGCCACTCCAAACATAGAGC-3′ and for β-actin CreER forward 5′-AACCTGGATAGTGAAACAGGGGC-3′ and reverse 5′-GGAACCGACTTGACGTAGCCAGC-3′. Male Sprague–Dawley rats for HSC isolation were purchased from Charles River Laboratories, UK. Pdgfrb-BAC-eGFP mice (provided by Professor Neil Henderson, University of Edinburgh, UK) on a C57BL/6 background carrying one copy of a BAC transgene expressing enhanced green-green fluorescent protein (eGFP) under the control of Pdgfrb regulatory elements have been described previously^18^. Animals were housed and maintained, and animal experiments performed under approval from the University of Manchester Ethical Review Committee and UK Government Home Office licence for animal research.

### HSCs, gene silencing, recombination and compound treatment

Under terminal anaesthesia, primary HSCs were isolated by perfusing pronase and collagenase through the liver via the portal vein, followed by isolation using Optiprep density gradients (Axis-Shield Diagnostics Ltd). For mouse, HSCs from two to five livers were combined per experimental replicate. For rat, sufficient HSCs were gained from single animals per experimental replicate. For primary human HSCs (isolated in the Liver Diseases Laboratory, Mount Sinai School of Medicine, NY, USA), in lieu of perfusion, liver tissue was finely dissected and incubated for 40 min at 37 °C in digestion buffer (0.5 mg ml^−1^ collagenase B, 0.2 mg ml^−1^ pronase)^43^. This was followed by isolation using Optiprep density gradients (Axis-Shield Diagnostics Ltd). Unless otherwise stated routine activation of primary HSCs was for 7 days by culture on tissue culture plastic with standard characterization for expected changes in phenotype and gene expression as an internal control^11^. Human LX2 cells were a gift from Professor Scott Friedman (Mount Sinai School of Medicine) 44.

All cells were cultured in monolayer at 5% CO_2_ and 37 °C in Dulbecco’s modified Eagle’s medium+L-glutamine, Na-pyruvate and antibiotics supplemented with fetal bovine serum; 10% for LX2 cells, or 16% for rat and mouse HSCs. Gene silencing in HSCs was carried out transiently using 10 nmol siRNA (Qiagen Supplementary Table 1 and for ITGA11 siRNA2: SMARTpool ON-TARGETplus Itga11 siRNA, Dharmacon/GE Healthcare) or scrambled control (Qiagen, UK) in Basic Nucleofector Kit T using programme U-25 on an Amaxa Nucleofector (Lonza).

For genetic recombination of integrin beta-1, HSCs isolated from *Itgb1*^fl/fl^ CreER^TM^± mice were treated with 100 nM tamoxifen (or the equivalent volume of ethanol vehicle for control) at the time of plating (day 0) and at media exchange on day 1 until day 5 (when media was next changed). For the disruption of YAP:TEAD complexes, 10 μM VP (Sigma, UK) or the equivalent volume DMSO vehicle control was included throughout media exchanges until cell collection. To investigate PAK signalling *in vitro*, 15 μM IPA3 (Tocris Bioscience) or the equivalent volume of DMSO vehicle control was added to the culture medium of activated HSCs on day 7 for a further 24 h before collecting for downstream analyses.

### Microarray analysis

In two replicate experiments RNA was isolated using the RNeasy kit (Qiagen) from HSCs extracted from *Itgb1*^fl/fl^ CreER+ mice: on the day of extraction (quiescent HSCs); or on day 8 (tamoxifen-treated ‘*Itgb1*-null’ or control-activated HSCs). The efficiency of integrin beta-1 recombination in tamoxifen-treated HSCs was 70–80% by qPCR (data not shown). RNA quality was assessed by Agilent Bioanalyser. Labelled RNA was hybridized to the Affymetrix Mouse Genome 430 2.0 Array platform according to Affymetrix protocols. Background correction, quantile normalization and quantification of gene expression were performed using RMA and differential expression was done with limma in Bioconductor (PMID: 15461798). For cluster analysis, differential gene expression was filtered by *P* value<0.05 and fold change>±1.5 in the ‘*Itgb1*-null’ versus control-activated HSC groups. In all, 915 genes organised into 7 clusters using a κ-means clustering algorithm (maxdView software (http://bioinf.man.ac.uk/microarray/maxd/); Fig. 2a and Supplementary Fig. S1). Clusters were analysed using Database for Annotation, Visualisation and Integrated Discovery (DAVID) version 6.7. Functional annotation clustering was carried out with medium stringency; functional clusters with an enrichment score>2 were selected, and proportionately represented by enrichment scores. Analysis of clusters 5 and 6 did not reveal any functional annotation clusters with an enrichment score>2. Clusters of interest were examined by Ingenuity Pathway Analysis (Ingenuity Systems, www.ingenuity.com).

### Histology, immunohistochemistry and immunocytochemistry

Tissue samples for histology and immunohistochemistry were fixed in 4% paraformaldehyde, embedded in paraffin wax and sectioned at 5 μm thickness. All livers were embedded in an identical orientation. Corresponding histology images within the manuscript are shown from the same liver lobe. To detect α-SMA, immunohistochemistry was carried out using anti α-SMA (M0851, DAKO, dilution: 1:100), the Mouse on Mouse basic detection kit (Vector laboratories) and Vectorstain Elite Avidin Biotin Complex (ABC) system (Vector laboratories). Antibody-complex colour reactions were developed with diaminobenzidine and counterstained with toluidine blue. The extent of scarring was assessed in livers using PSR staining (Sigma). Quantification of PSR and α-SMA staining was determined by morphometric analysis. Images were acquired from stained slide sections using the 3D Histech Pannoramic 250 Flash II slide scanner. Ten regions which included all three lobes of the liver (100 × total magnification), were selected at random and analysed using Adobe Photoshop. The Colour Range tool was used to select stained pixels (red for PSR and brown for α-SMA); the number of selected pixels was recorded and expressed as a fraction of the total number of pixels, averaged across the 10 different regions per section. All quantification was carried out blinded, without prior knowledge of sections or treatment/control group. For immunocytochemistry, cells were cultured on glass chamber slides, fixed in 4% paraformaldehyde and incubated with the following primary antibodies: anti-integrin beta-1 (MAB1997, Millipore, dilution: 1:200), anti α-SMA (SMA-R-7-CE, Leica Biosystems, dilution: 1:100), anti-SOX9 (AB5535, Millipore, dilution: 1:1,000), anti-YAP (Sc-101199, Santa Cruz, dilution: 1:200) and anti-PMYL9 (3671, Cell Signalling, dilution: 1:50). For all immunofluorescence secondary antibodies were 488 or 594 Alexa Fluors (Molecular Probes, Invitrogen) raised against the appropriate species (dilution: 1:1,000). For G- and F-actin, cells were fixed in 3.7% paraformaldehyde, permeabilized in 0.1% Triton X-100 in phosphate-buffered saline (PBS) and incubated with Alexa Fluor 594 DNase I to detect G-actin (Life Technologies) followed by Alexa Fluor 488 Phalloidin to detect F-actin (Life Technologies).

### Gene protein and co-immunoprecipitation analysis

For quantitative reverse-transcription–polymerase chain reaction (qRT–PCR), RNA was extracted from tissue and cells using RNeasy (Qiagen) and converted to cDNA using a High-Capacity RNA-to-cDNA kit (Life Technologies). Intron-spanning primers were used wherever possible (Supplementary Table 2). *GusB* and *ActinB* were used in combination as standardizing ‘housekeeping’ genes for SYBR Green (Primer Design, UK) gene expression assays. For *in vivo* activated HSCs *Pdgfrb* was used as the reference gene, as recommended by Mederacke *et al*.^34^. Changes in mRNA expression were calculated using ^ΔΔ^C_T_ methodology. Western blotting was carried out using standard techniques and protein detected using the following primary and secondary antibodies: anti-integrin beta-1 (MAB1997, Millipore, dilution: 1:1,000), anti-α-SMA (SMA-R-7-CE, Leica Biosystems, dilution: 1:100), anti-COL1 (1310-01, Southern Biotech, dilution:1:1,000), anti-SOX9 (AB5535, Millipore, dilution: 1:5,000), anti-MYL9 (3672, Cell Signalling, dilution: 1:500), anti-YAP (Sc-271134, Santa Cruz, dilution: 1:1,000), anti-PMYL9 (#3671P, Cell Signalling, 1:500), anti-PAK-1 (#2602, Cell Signalling, dilution: 1:1,000), anti-PAK-2 (#2604, Cell Signalling, dilution: 1:1,000), anti-PAK-3 (#2609, Cell Signalling, dilution: 1:1,000), anti-ITGA11 (MAB4235, R&D Systems, dilution: 1:500), anti-integrin alpha-V (611012, BD Transduction Laboratories, dilution: 1:500), anti-Caspase (#9662S, Cell Signalling, dilution: 1:1,000), anti-β-actin horseradish peroxidase conjugate (A3854, Sigma, dilution: 1:50,000) and species-specific horseradish peroxidase conjugated secondary antibodies (GE Healthcare). For immunoprecipitation of endogenous protein complexes, activated rat HSCs were lyzed in NETN buffer (100 mM NaCl, 20 mM Tris-Cl, 0.5 mM EDTA, 0.5% (v/v) Nonidet P-40, 1 × EDTA free Protease Inhibitors, and 1 × Inhibitor Cocktails 1 and 2). Cell lysate was pre-cleared with magnetic protein-G-coated beads (New England Biolabs) and rat IgG (purified rat IgG, R&D Systems) to avoid non-specific protein binding during the immunoprecipitation. To immunoprecipitate integrin beta-1 protein complexes, magnetic protein-G beads were coated with anti-integrin beta-1 (Millipore) or control IgG antibody (R&D Systems) and incubated with cell lysate. Integrin beta-1 protein complexes were eluted from beads by magnetic separation. Total supernatant and immunoprecipitated lysates were analysed by immunoblotting using anti-integrin beta-1 (Millipore), anti-ITGA11 (R&D Systems) and ITGA3 (AB1920, Millipore; data not shown). Full-length uncropped blots are provided in Supplementary Fig. 14.

### Chromatin immunoprecipitation

For chromatin immunoprecipitation, HSCs were fixed in 1% formaldehyde for 10 min, and chromatin prepared and sheared by sonication to generate 200–1,000 bp fragments. Sheared chromatin was incubated overnight at 4 °C with 3 μg of anti-YAP (Sc-271134 X, Santa Cruz) or control IgG antibody (R&D Systems) and magnetic protein-G-coated beads (pre-cleared with sheared salmon sperm and BSA). Antibody-bead complexes were washed three times in each of the following buffers: Buffer 1 (20 mM Tris-HCl pH 8.1, 2 mM EDTA, 50 mM EDTA, 0.1% SDS, 1% Triton X-100), Buffer 2 (10 mM Tris-HCl pH 8.1, 1 mM EDTA, 250 mM LiCl, 1% NP-40, 1% Deoxycholate), and TE Buffer (10 mM Tris-HCl pH 8.1, 1 mM EDTA). Protein-DNA complexes were eluted, crosslinks reversed and DNA purified using MinElute columns (Qiagen). DNA was analysed by PCR (Supplementary Table 2).

### Cell migration

Activated HSCs were cultured in 24-well plates at a density of 5,000 cells per well. Single-cell tracks were acquired using live-cell imaging at 10 min intervals over a 24 h period (AS MDW live cell imaging system, Leica). Multiple position imaging was gathered using Image Pro 6.3 (Media Cybernetics Ltd) and a Coolsnap HQ (Photometrics) camera with a Z optical spacing of 0.2 μm. Cell movement over the 24 h period was analysed using ImageJ software with the MTrackJ plug-in. Total length of the cell track (in μm) was generated for multiple cells (details are provided in the figure legends), averaged and normalized to control. Where individual cell migration tracks are shown, the co-ordinates at 10 min intervals were plotted relative to each cell’s starting point (set at 0,0).

### Cell proliferation

Cell proliferation was assessed using BrdU incorporation. Activated HSCs were plated onto chamber slides (5,000 cells per well) and incubated with 30 μM BrdU (Sigma, UK) for 4 h. Cells were fixed and processed for immunofluorescence counterstained with DAPI. BrdU was detected using sheep anti-BrdU primary antibody (ab1893; Abcam, UK, 1:1,000) and donkey anti-sheep fluorescein isothiocyanate (FITC)-conjugated secondary antibody (Abcam, 1:500). Images were captured from two areas of each chamber well at × 100 total magnification (Axio AI Imager and Axiovision software). BrdU^+^/DAPI^+^ nuclei were counted using Adobe Photoshop CS4 software and expressed as a percentage of the total DAPI^+^ population.

### Collagen gel contraction assay

Because of the large quantity of cells required for a type I collagen gel contraction assay, *Itgb1*fl/fl CreER+HSCs were activated in culture for 7 days before incubation with 100 nM tamoxifen (‘Itgb1-null’) or ethanol vehicle (‘Control’) for a further 48 h. Inactivation of integrin beta-1 was confirmed as described earlier (in addition to which extra cell migration assays were performed (Supplementary Fig. S2)). The collagen gel contraction assay was performed based on a previous protocol^19^. A total of 75,000 control or ‘*Itgb1*-null’ HSCs were suspended in culture medium mixed with type I collagen, and aliquoted into a 12-well culture plate. Once polymerised, gels were detached from the sides of the well, imaged (time zero; Biorad Chemidoc under white light), and re-imaged after 24 h free-floating culture at 37 **°**C. Gel contraction was calculated using ImageJ software as the change in gel surface area between time zero and 24 h. Results are expressed as a percentage of the contraction achieved by control-activated HSCs.

### Cell viability

Cell viability was assed using the 3-(4-5-dimethylthiazol-2-yl)-2,5-diphenyl tetrazolium bromide (MTT) assay (Sigma) as per the manufacturer’s instructions. A total of 25,000 activated HSCs, assessed to be growing in log phase, were plated per well of a 6-well plate. Cell viability was detected at 560 nm absorbance in the presence of MTT solvent.

### Plasmids and luciferase assay

Full-length human *YAP1* cDNA was sub-cloned from a pcDNA Flag-YAP1 vector (plasmid 18881, Addgene, Cambridge, USA)^45^ into the *Kpn*I(5’)/*Xba*I(3’) sites of pcDNA3.1zeo+ (Life Technologies) using forward (5′-CCCGGTACCATGGATCCCGGGCAGCAGCCG-3′ and reverse 5′-TCGTCTAGACTATAACCATGTAAGAAAGCT-3′) primers. The sequence containing the TEAD-binding site of human *MYL9* gene was cloned into *Kpn*I/*Xho*I sites of the pGL3-promoter reporter vector (Promega, UK) from human genomic DNA using forward (5′-AGTGGTACCGTCCCAGTTCCC-3′) and reverse 5′-CATGCTCGAGCATTTGTGTGGGGATAC-3′) primers. The mutated *MYL9* pGL3-promoter vector was generated by deleting the 5′-CATTCC-3′ conserved TEAD-binding motif. LX2 cells were transfected with the reporter plasmids plus pRL-TK (Promega) and the YAP expression vector or control empty vector using Transfast reagent (Promega). Luciferase activity in cell lysates was detected using the Dual-Luciferase reporter assay system (Promega). Relative reporter units were normalized to *Renilla* luciferase activity from the pRL-TK vector. All results are shown compared with the relevant empty vector controls.

### CCl_4_-induced fibrosis

Mice were given intraperitoneal (i.p.) injections of 2 μl per g body weight CCl_4_ (Sigma) at ratio of 1:3 by volume in olive oil (Sigma) or olive oil alone (control) twice weekly for 8 weeks.

For the VP (VP) experiments, fibrosis was induced by CCl_4_ injections for 6 weeks with VP treatment for the final 3 weeks. VP was dissolved in 10% DMSO in PBS to a concentration of 10 mg ml^−1^. Ten microlitre per g body weight (100 mg VP/kg bodyweight) was injected i.p. three times per week (on alternate days to CCl_4_ or olive oil). Control mice were injected with DMSO. This created four treatment groups: olive oil+DMSO (*n*=5); olive oil+VP (*n*=3); CCl_4_+DMSO (*n*=3); and CCl_4_+VP (*n*=4).

Treatment with IPA3 (Tocris Bioscience) or DMSO (Sigma) occurred during the final 4 weeks. IPA3 was dissolved in DMSO to a concentration of 4 μg μl^−1^; 1 μl per g body weight (4 mg IPA3/kg bodyweight) was injected i.p. three times per week (on alternate days to CCl_4_ or olive oil injections). Control mice were injected with DMSO. This created four treatment groups: olive oil+DMSO (*n*=5); olive oil+IPA3 (*n*=5); CCl_4_+DMSO (*n*=5); and CCl_4_+IPA3 (*n*=5).

### BDL induction of fibrosis

Under anaesthesia the bile duct was exposed and ligated using two ligatures. Sham-operated mice underwent the same procedure but without tying the ligatures. BDL mice developed cholestasis and associated fibrosis over a 14-day period.

As for CCl4, VP was dissolved in 10% DMSO in PBS to a concentration of 10 mg ml^−1^. 10 μl per g body weight (100 mg VP/kg bodyweight) and was injected every 48 h starting on day 7 after BDL. This created four treatment groups: sham+DMSO (*n*=8), sham+VP (*n*=4), BDL+DMSO (*n*=7), BDL+VP (*n*=5).

For IPA3, at equal intervals during the second post-operative week, mice were given three i.p. injections of 4 μg μl^−1^ IPA3 (1 μl per g body weight) in DMSO, or DMSO alone. This created four treatment groups: sham+DMSO (*n*=4); sham+IPA3 (*n*=4); BDL+DMSO (*n*=8); and BDL+IPA3 (*n*=7).

At the end of all experiments, animals were killed, blood taken for liver function biochemistry, liver and body weight recorded and livers processed for histology.

### Isolation of *in vivo* activated HSCs

*In vivo* activated mouse HSCs were induced and isolated exactly as described in the published study by De Minicis *et al*.^33^. In brief, mice were injected i.p. on 4 occasions at 2-day intervals with 2 μl per g body weight CCl_4_ (Sigma) at a ratio of 1:3 by volume in olive oil (Sigma) or olive oil alone (control). HSCs were then isolated by liver perfusion and density gradient as described above and prepared for qPCR analysis as described previously^33^,^34^. In addition, *in vivo* activated GFP-labelled HSCs were also extracted from Pdgfrb-BAC-eGFP reporter mice following 6-weeks CCl_4_-induced fibrosis or olive oil control for analysis by fluorescence-activated cell sorting (FACS) as previously described^18^. The GFP-positive cells were resuspended in FACS buffer (PBS supplemented with 2% FCS and 2 mM EDTA). Following a 10 min block with 10% mouse serum and CD16/32, cells were incubated with a phycoerythrin-conjugated anti-integrin beta-1 (12-0291, 1:200) or isotype control (12-4888, 1:200; both from eBioscience) at 4 °C for 20 min in the dark^18^. Cells were washed, resuspended in FACS buffer and single, live, GFP-positive HSCs analysed on a Becton Dickinson LSR Fortessa.

### Hydroxyproline assay

Hydroxyproline content was measured using the QuickZyme Biosciences Total Collagen Assay and Total Protein Assay kits (QuickZyme Biosciences, The Netherlands). Liver tissue was hydrolysed from 10 × 10 μm thick paraffin-embedded sections in HCl at 95 °C for 20 h as per the manufacturer’s instructions. Hydroxyproline content was determined by absorbance at 570 nm against standard kit controls and expressed per total amount of protein assessed in the sample.

### Statistical analysis

Statistical significance was determined by two-tailed Student’s *t*-test. All experiments were carried out three times or more (*n*=3).

### Data availability

Microarray data that support the findings of this study have been deposited in ArrayExpress with the accession code E-MTAB-4810. The authors declare that all other data supporting the findings of this study are available within the article and its Supplementary Information files, or from the corresponding author on request.

## Additional information

**How to cite this article**: Martin, K. *et al*. PAK proteins and YAP-1 signalling downstream of integrin beta-1 in myofibroblasts promote liver fibrosis. *Nat. Commun.* 7:12502 doi: 10.1038/ncomms12502 (2016).

## Supplementary Material

Supplementary Information Supplementary Figures 1-14 and Supplementary Tables 1 & 2.
