# Supplementary material for: PAK proteins and YAP-1 signalling downstream of integrin beta-1 in myofibroblasts promote liver fibrosis
Source: Nat Commun. 2016 Aug 18;7:12502. doi: 10.1038/ncomms12502 (PMC4992158; doi:10.1038/ncomms12502)
Supplement: Supplementary Information — Supplementary Figures 1-14 and Supplementary Tables 1 & 2. [file ncomms12502-s1.pdf]

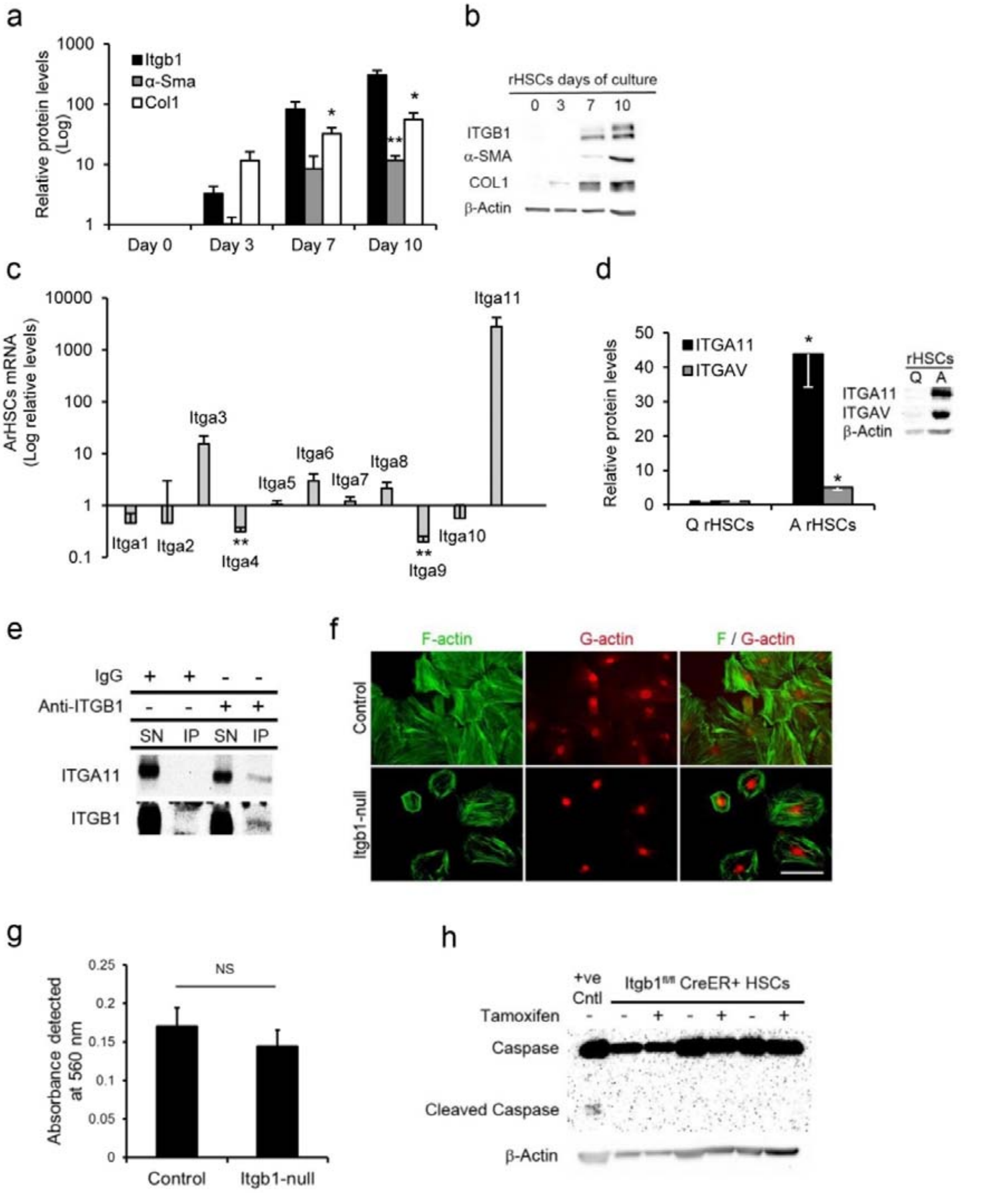

Supplementary Figure 1 ITGB1 and ITGA11 increase with evidence for heterodimers following HSC activation. (**a**) Time course of rat HSC activation indicated by the detection of α-SMA and COL1 (log scale). (**b**) Representative immunoblot underlying (**a**). The double band for ITGB1 is consistent with detection of precursor 115 KDa and mature 130 KDa forms. (**c**) qRT-PCR analysis of integrin alpha subunits in activated rat HSCs (ArHSCs) relative to their quiescent counterparts (log scale). (**d**) Quantification and representative immunoblot (inset) of ITGA11 and ITGAV protein levels in ArHSCs compared to their quiescent (Q) counterparts. (**e**) Coimmunoprecipitation studies showing evidence for ITGA11B1 heterodimer complexes in activated rat HSCs. Representative immunoblot of two independent experiments is shown. SN = Supernatant and IP = Immunoprecipitation. (**f**) Immunofluorescence following Itgb1 loss ('Itgb1-null') in activated mouse HSCs ('Control') for F-actin (green; located along stress fibres) and G-actin (red; distributed in the nucleus and cytoplasm of control cells). Following loss of Itgb1 in activated mouse HSCs F-actin is diminished and cells are much more rounded. Scale bars 50μm. (**g**) MTT assay for cell viability following Itgb1 loss ('Itgb1-null') in activated mouse HSCs ('Control'). (**h**) The detection of apoptosis by Caspase immunoblot is unaltered following Itgb1 inactivation (+Tamoxifen) in activated mouse HSCs (-Tamoxifen). The positive control (+ve Cntl) is lysate prepared from UV-treated activated rat HSCs. All experiments are

n=3 to 6 unless otherwise indicated. Two-tailed unpaired *t*-test was used for statistical analysis. Data are shown as means ± s.e.m.. *P<0.05, **P<0.01.

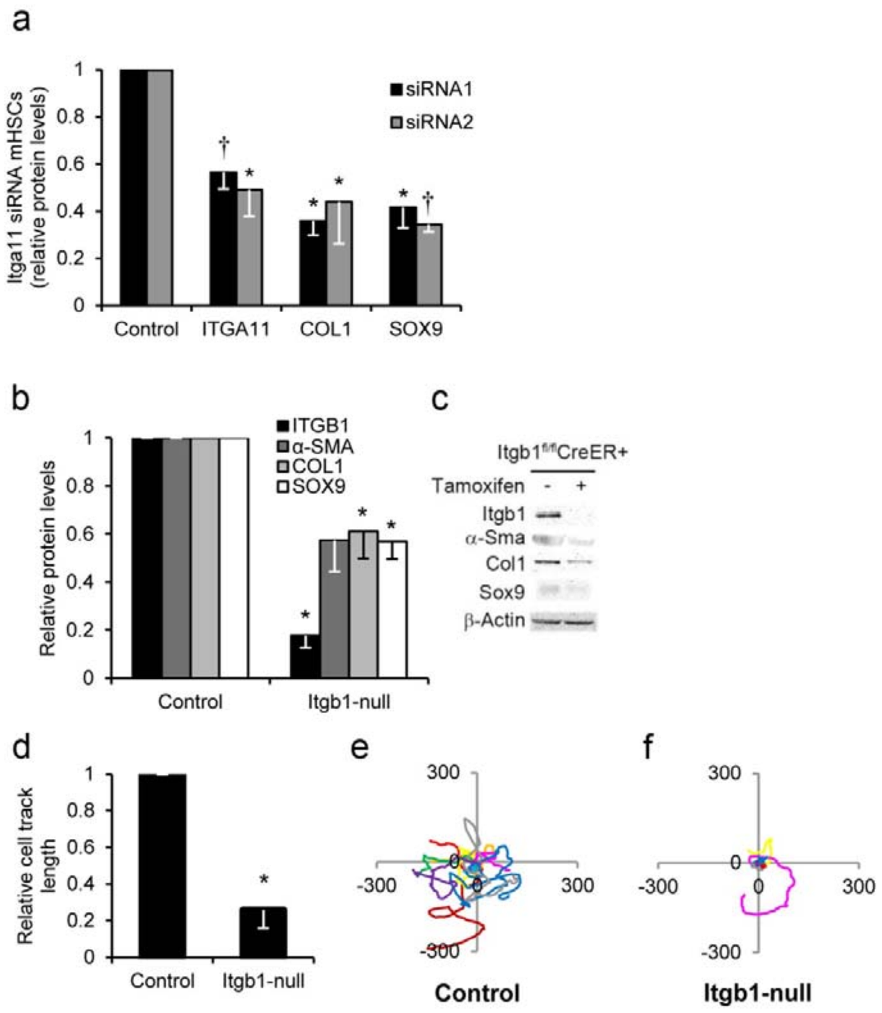

Supplementary Figure 2 Abrogation of either ITGA11 or ITGB1 causes loss of activated HSC / myofibroblast characteristics. (**a**) Quantification of COL1 and SOX9 protein levels following moderate ITGA11 knockdown by siRNA1 (n=8) or siRNA2 (n=3) in activated mouse HSCs expressed relative to scrambled control (immunoblots for ITGA11 siRNA knockdown are available in Supplementary Figure 8). (**b, c**) Quantification and example immunoblots following loss of Itgb1 ('Itgb1-null', n=4) in mouse HSCs already activated for 7 days showing decrease in protein levels for α-SMA, COL1 and SOX9. (**d-f**) Migration of activated mouse HSCs ('Control') over 24h versus migration of equivalent HSCs in which Itgb1 had been recombined only after full activation (track length, μm; n=3 biological replicate experiments) (**d**). Individual tracks for a small subset of cells are shown in different colours for one experiment in (**e**) and (**f**). Two-tailed unpaired *t*-test was used for statistical analysis. Data are shown as means ± s.e.m.. *P<0.05, †P<0.005.

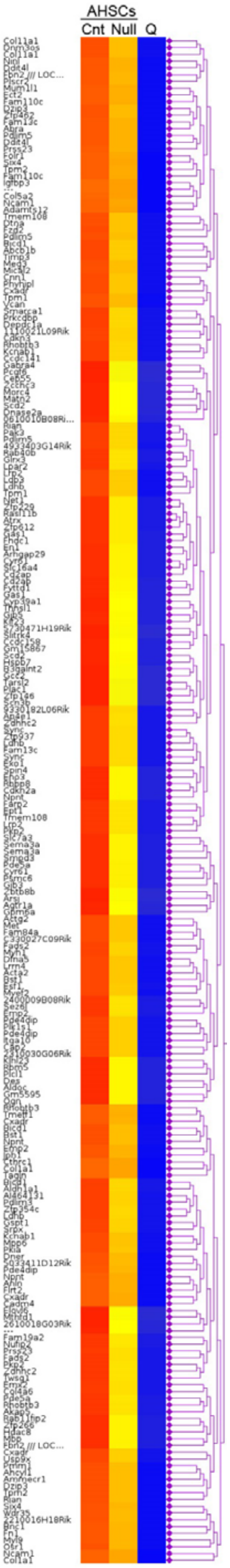

Supplementary Figure 3 Hierarchical clustering and heatmap for Cluster 3 (Fig. 2a) with full gene list. Colour indicates upregulated (red), downregulated (blue) and intermediate (yellow) gene expression for activated (A) mouse HSCs (Cnt) and following the loss of Itgb1 (Null). The data here depict the mean signal of replicate microarrays with quiescent (Q) HSCs shown for comparison.

Supplementary Figure 4 Functional annotation by gene ontology for enrichment in Cluster 7 from Fig. 2a. Proportions are shown in (**a**). Individual categories and the genes underlying them are shown in (**b**).

a

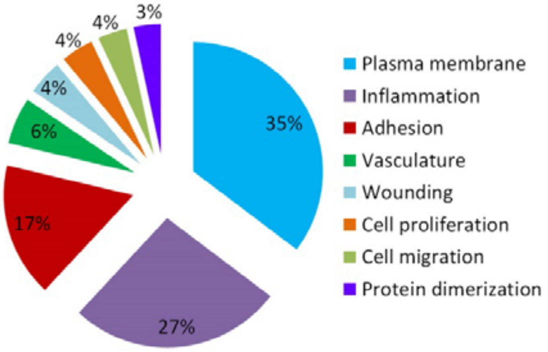

b

Cluster 7: Functional annotation clustering (Enricment Score >2.0)

Plasma membrane: combined Enrichment Score (8.82, 6.05, 4.99, 2.72) = 22.58

CORE CATEGORY	P VALUE	GENE SYMBOL
External side of plasma membrane	2.9 E-12	*Clec7a, Cd22, Cd274, Cd28, Cd48, Cd83, Vwf, Ctsb, H2-D1, H2-K1, H2-Qb, H60a, Itga4, ItgaX, Il7r, Il2rg, Ptprc, Robo4, Stab2*
Cell surface	2.6 E-10	*Clec7a, Cd22, Cd36, Cd274, Cd28, Cd48, Cd83, Vwf, Ctsb, H2-D1, H2-K1, H2-Qb, H60a, Itga4, ItgaX, Il7r, Il2rg, Ptprc, Robo4, Stab2*
Membrane	1.8 E-6	*Abcc3, Arap3, Blnk, Clcc7a, Cd22, Cd274, Cd28, Cd300A, Cd36, Cd48, Cd83, Eltd1, Gpr116, Gpr97, Notch1, Slamf8, St8sia4, St8sia6, Acer2, Aqp1, Arrb1, Bace2, Cdh5, Cxcr4, Cldn4, F8, Csf1r, Csf2rb, CybB, Dock2, Evi2b, Entpd1, Eng, Emcn, Fam26f, Fmo1, Folr2, HpsE, H2-D1, H2-K1, H2-Qb, Hvcn1, Hsd11b1, Itga4, ItgaX, Iigp1b, Il7r, Klra3, Lrre33, Lyve1, Lhfpl2, Ly9, Lcp1, Laptm5, Myct1, Nrp2, ParvB, Ppap2b, Prex1, Pde2a, Pik3ap1, Pld4, Plvap, Pecam1, Plxnc1, Plxnd1, Kcnn4, Cyp4b1, Bcl2, Il2rg, Ptger4, Prkcb, Ptprb, Ptprc, Rsad2, Rtp4, Selplg, Sema4d, Stab2, Sorl1, Slc40A1, Slc43a2, Slc9a9, Spns2, Stab1, Tspan13, Tmem140, Tmem204*

Inflammation: combined Enrichment Score (3.81, 3.47, 3.11, 2.11, 2.32, 2.2) = 17.02

CORE CATEGORY	P VALUE	GENE SYMBOL
Cell activation	1.6 E-10	*Blnk, Cd28, Cd48, Fyb, Ikzf1, Vwf, Was, Cxcr4, Dock2, Entpd1, H60a, ItgaX, Il7r, Lcp1, Plcg2, Bcl2, Prkcb, Sykb, Vav1*
Hemopoiesis	3.3 E-6	*Cepba, Cd28, Ikzf1, Tal1, Epas1, Hcls1, Irf8, Il7r, Plcg2, Bcl2, Prkcb, Sykb, Vav1*
Cell proliferation	5.4 E-3	*Cxcr4, Dock2, Fabp7, Hhex, ItgaX, Irf6, Il7r, Bcl2, Prkcb*
Positive regulation of immune system processes	4.9 E-8	*Clec7a, Cd28, Cd83, Ikzf1, Sash3, H2-K1, H2-Qb, H60a, Il7r, Masp1, Plcg2, Il2rg, Ptpn6, Ptprc, Sykb*

Adhesion: combined Enrichment Score (7.58, 3.14) = 10.72

CORE CATEGORY	P VALUE	GENE SYMBOL
Cell adhesion	1.5 E-9	*Clec7a, Cd22, Cd36, Vwf, Cdh5, Cdn4, F8, Cyfip2, Dpt, Eng, Emcn, Fermt3, Itga4, ItgaX, Klra3, Lyve1, Ly9, Nrp2, Parvb, Pcam1, Bcl2, Ptprc, Selplg, Stab2, Stab1, Tgfbi*

Vasculature: Enrichment Score = 3.74

CORE CATEGORY	P VALUE	GENE SYMBOL
Blood vessel development	6.9 E-5	*Egfl7, Notch1, Zmiz1, Cdh5, Cxxr4, Eng, Emcn, Epas1, Itga4, Ppap2b, Plxnd1, Robo4*

Wounding: Enrichment Score = 2.8

CORE CATEGORY	P VALUE	GENE SYMBOL
Defense response	1.0 E-4	*Clec7a, Dhx58, Ccl11, Ccl5, F8, H2-D1, H2-K1, H2-Qb, Irf8, Masp1, Bcl2, Penk, Ptpn6, Ptprc, Rsad2, Stab1*

Cell proliferation: Enrichment Score = 2.59

CORE CATEGORY	P VALUE	GENE SYMBOL
Regulation of cell proliferation	7.1 E-5	*Cepba, Cd274, Cd28, Notch1, Zmiz1, Sash3, Cdh5, Dpt, Hcls1, Hhex, Irf6, Nr5a2, Bcl2, Odc1, Ptpn6, Ptprc, Slfn2, Sykb,*

Cell migration: Enrichment Score = 2.34

CORE CATEGORY	P VALUE	GENE SYMBOL
Regulation of cell migration	3.2 E-3	*Arap3, Egfl7, Cxcr4, Pecam1, Bcl2, Robo4*

Protein dimerization: Enrichment Score = 2.16

CORE CATEGORY	P VALUE	GENE SYMBOL
Protein dimerization activity	2.5 E-3	*Cepba, Ikzf1, Vwf, Atf3, Ctse, Eng, Epas1, Masp1, Nfe2l2, Bcl2*

a

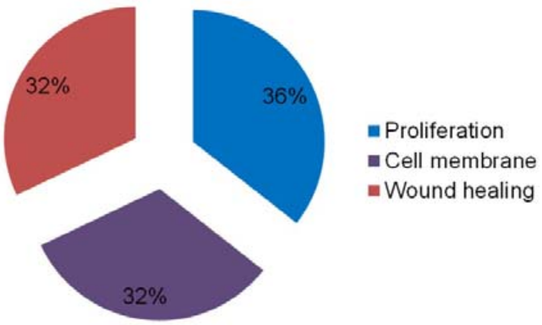

b

Cluster 2: Functional annotation clustering (Enrichment Score >2.0)

	CATEGORY	P VALUE	GENE SYMBOL
**Proliferation** (Enrichment: 2.31)	Cell proliferation	1.5 E-5	*Ccnd1, Gng2, Itgb2, Il7r, Ncf1, Tbx1, Pdgfb, Slc11a1*
	Cell activation	1.5 E-4	*Fyb, Entpd1, Itgb2, Il7r, Myo1f, Plek, Slc11a1*
	Cell surface	1.9 E-2	*Cd93, Itgb2, Il7r, Slc11a1, Slco3a1*
**Cell membrane** (Enrichment: 2.1)	Glycoprotein	9.3 E-5	*Cd93, Mamdc2, Sel1l3, Chl1, Csf2rb2, Entpd1, Itgb2, Il7r, Lgals3bp, Psap, Ptpro, Ptpre, P2rx4, Gpr137b, Nn1, Pdgfb, Slc11a1, Slc15a3, Slco3a1, Slco4a1, Tslp, Tfpi2, Trpv2, Tnfrsf21*
	Membrane	8.6 E-4	*Atp13a2, Adap2, Cd93, Ehd4, Rasa4, Sel1l3, Tyrobp, Chl1, Csf2rb2, Daglb, Entpd1, Grina, Gng2, Itgb2, Il7r, Mcoln2, Pik3r5, Ank, Ptpre, P2rx4, Gpr137b, Nn1, Pdgfb, Slc11a1, Slc15a3, Slco3a1, Slco4a1, Stxbp5, Trpv2, Tnfrsf21, Vps18*
	Signal	7.8 E-3	*Cd93, Mamdc2, Tyrobp, Chl1, Csf2rb2, Itgb2, Il7r, Lgals3bp, Psap, Ptpro, Ptpre, Cxcl5, Nn1, Pdgfb, Tslp, Tfpi2, Tnfrsf21*
**Wound healing** (Enrichment: 2.08)	Response to wounding	1.3 E-4	*Entpd1, Itgb2, Ncf1, Pparg, Plek, Cxcl5, Slc11a1, Tfpi2*

Supplementary Figure 5 Functional annotation by gene ontology for enrichment in Cluster 2 from Fig. 2a. Proportions are shown in (**a**). Individual categories and the genes underlying them are shown in (**b**).

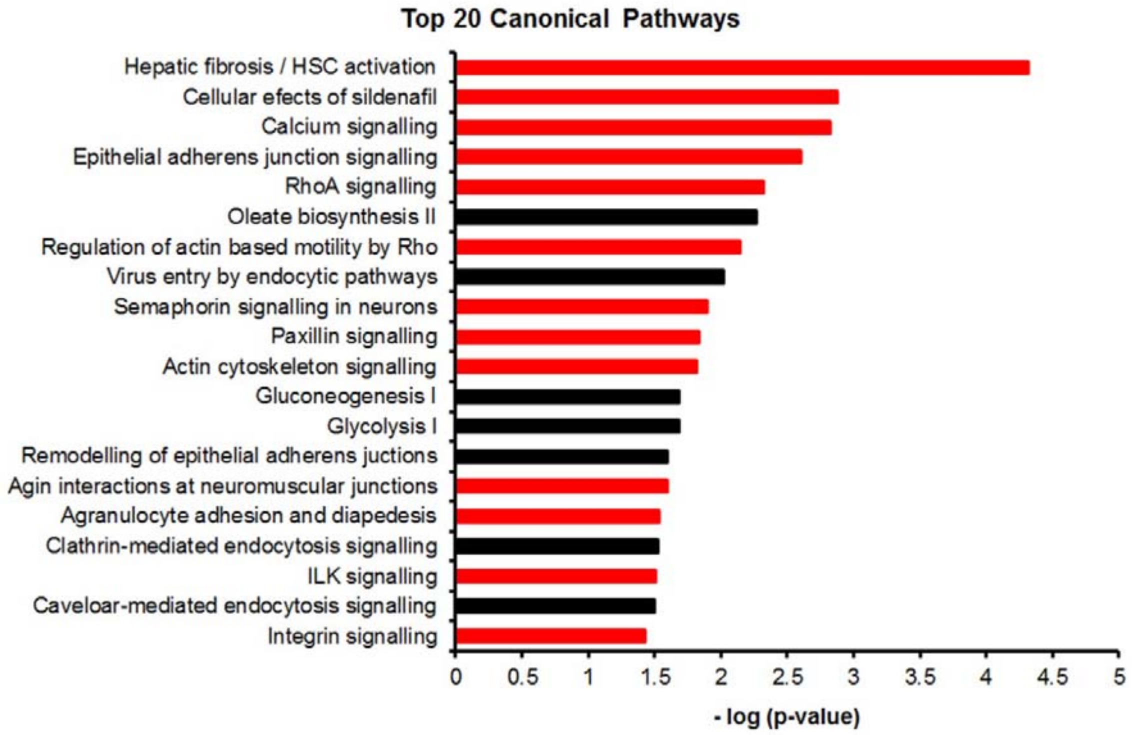

Supplementary Figure 6 Top 20 canonical pathways represented by genes listed in Cluster 3 (Fig. 2a-c) following Ingenuity Pathway Analysis. Pathways were ranked by the negative log of P-values calculated by Fisher's exact test for gene enrichment. Pathways highlighted in red contain either '*Myl9'* and/or '*Pak'* as terms.

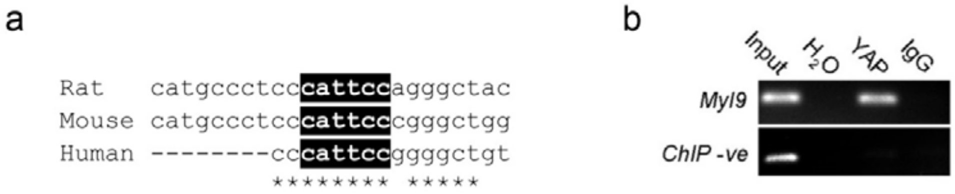

Supplementary Figure 7 YAP binds to a TEAD motif in the *MYL9* gene. (**a**) Alignment of *MYL9* 3’ UTR shows the conserved TEAD-binding motif (core motif shown in black). Conservation is indicated by asterisks (*). (**b**) Representative ChIP assay (n=3) for TEAD-binding element from (**a**) showing enrichment for the TEAD co-factor, YAP, in chromatin prepared from activated rat HSCs. Negative control is immunoglobulin (IgG) and positive control is input (diluted 10-fold).

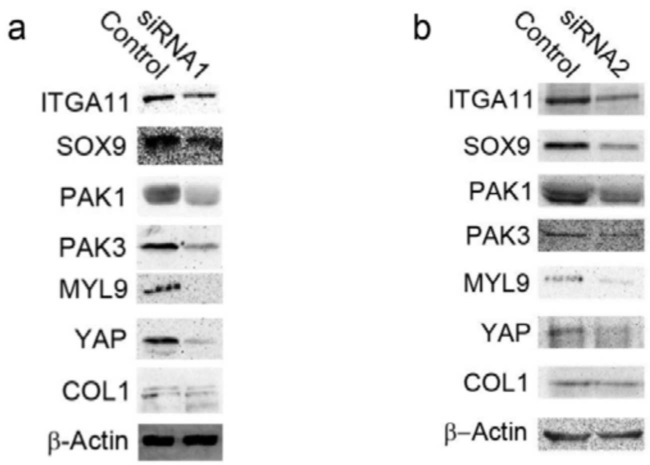

Supplementary Figure 8 Representative immunoblots for all ITGA11 siRNA knockdown quantified in Figures 3 & 5 and Supplementary Figure 2. Abrogation of ITGA11 is by two independent siRNAs, siRNA1 (**a**) and siRNA2 (**b**). Please see raw data files for full immunoblots containing molecular weight markers.

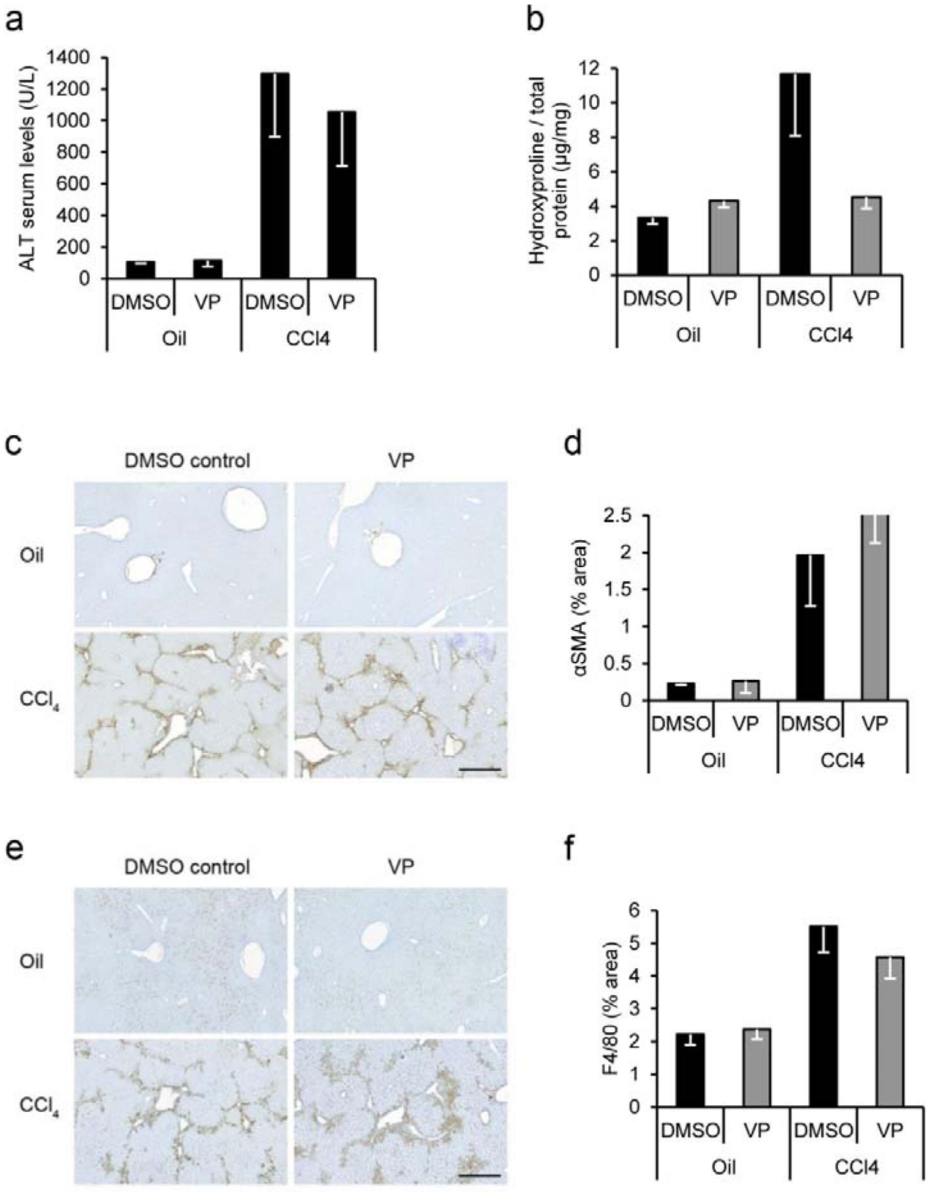

Supplementary Figure 9 Characterisation of mouse livers following verteporfin (VP) treatment in CCl_4-induced liver fibrosis. (**a**) VP treatment did not affect serum alanine aminotransferase (ALT) levels compared to the DMSO control group. (**b**) Hydroxyproline quantification seemed lessened in the VP-treated CCl_4 liver fibrosis group but did not reach statistical signficance. (**c-f**) VP treatment did not significantly alter myofibroblast cell numbers quantified by αSMA staining (c-**d**) or inflammatory cells (predominantly macrophages), quantified by F4/80 staining (e-**f**) compared to DMSO control group. Two-tailed unpaired *t*-test was used for statistical analysis. Data are shown as means ± s.e.m..

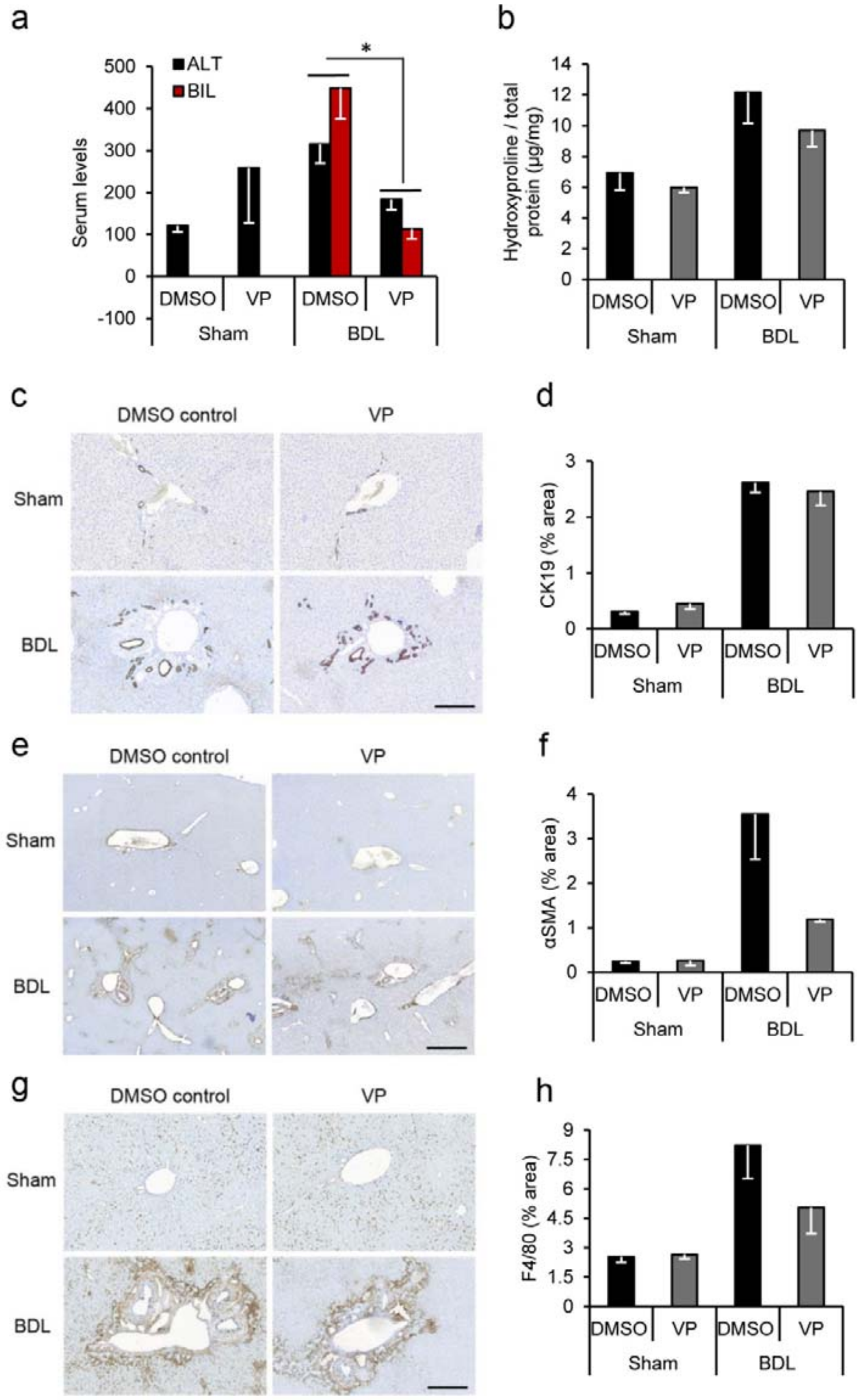

Supplementary Figure 10 Characterisation of mouse livers following VP treatment in BDL-induced liver fibrosis. (**a**) VP treatment in BDL improved liver function as indicated by lower serum alanine aminotransferase (ALT) and bilirubin (BIL) levels compared to the DMSO control group. (**b**) Hydroxyproline quantification was not statistically lowered in VP-treated BDL liver fibrosis. (**c-h**) VP treatment did not significantly alter ductal hyperplasia as quantified by the surface area covered by CK19 positive ducts (**c-d**), although there was a trend to lower myofibroblast cell numbers, quantified by αSMA staining, (**e-f**) and inflammatory cells (predominantly macrophages) quantified by F4/80 staining (**g-h**) compared to DMSO control group. Two-tailed unpaired *t*-test was used for statistical analysis. Data are shown as means ± s.e.m.. *P<0.05.

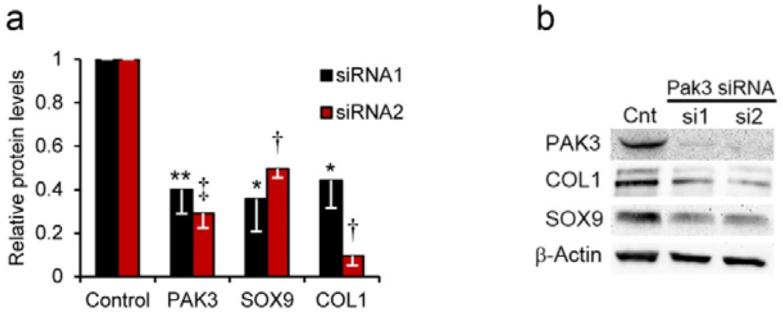

Supplementary Figure 11 Abrogation of PAK3 by two independent siRNAs in activated mouse HSCs. Quantification (**a**) and representative immunoblot (**b**) showing reduced levels of pro-fibrotic proteins / activated HSC markers following abrogation of PAK3 relative to control scrambled siRNA. Two-tailed unpaired *t*-test was used for statistical analysis. Data are shown as means ± s.e.m.. *$P<0.05$, **$P<0.01$, †$P<0.005$, ‡$P<0.001$; n=3.

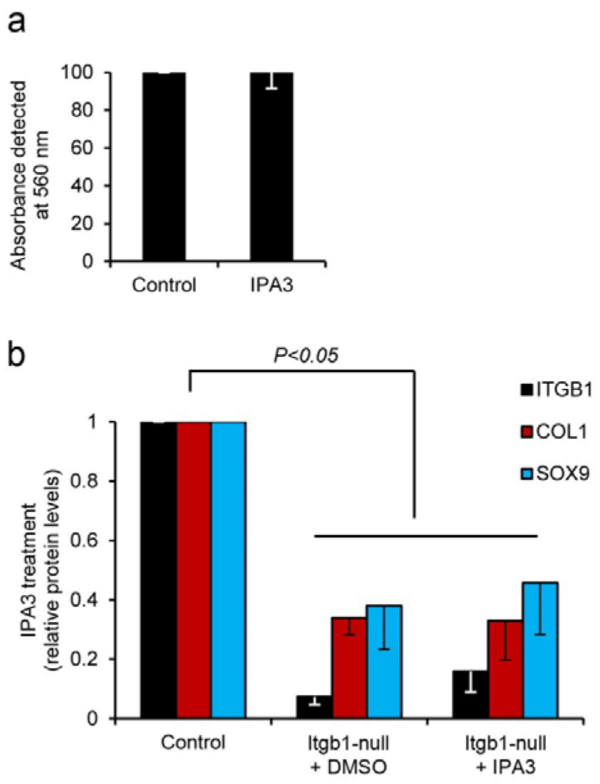

Supplementary Figure 12 IPA3 treatment does not affect cell viability of activated HSCs or alter fibrotic markers in Itgb1-null activated HSCs. (**a**) MTT assay for cell viability following IPA3 treatment in activated mouse HSCs. Control is DMSO treated cells. (**b**) IPA3 treatment does not alter levels of profibrotic markers, COL1 and SOX9, in Itgb1-null activated HSCs compared to DMSO treated cells. Two-tailed unpaired *t*-test was used for statistical analysis. Data are shown as means ± s.e.m..

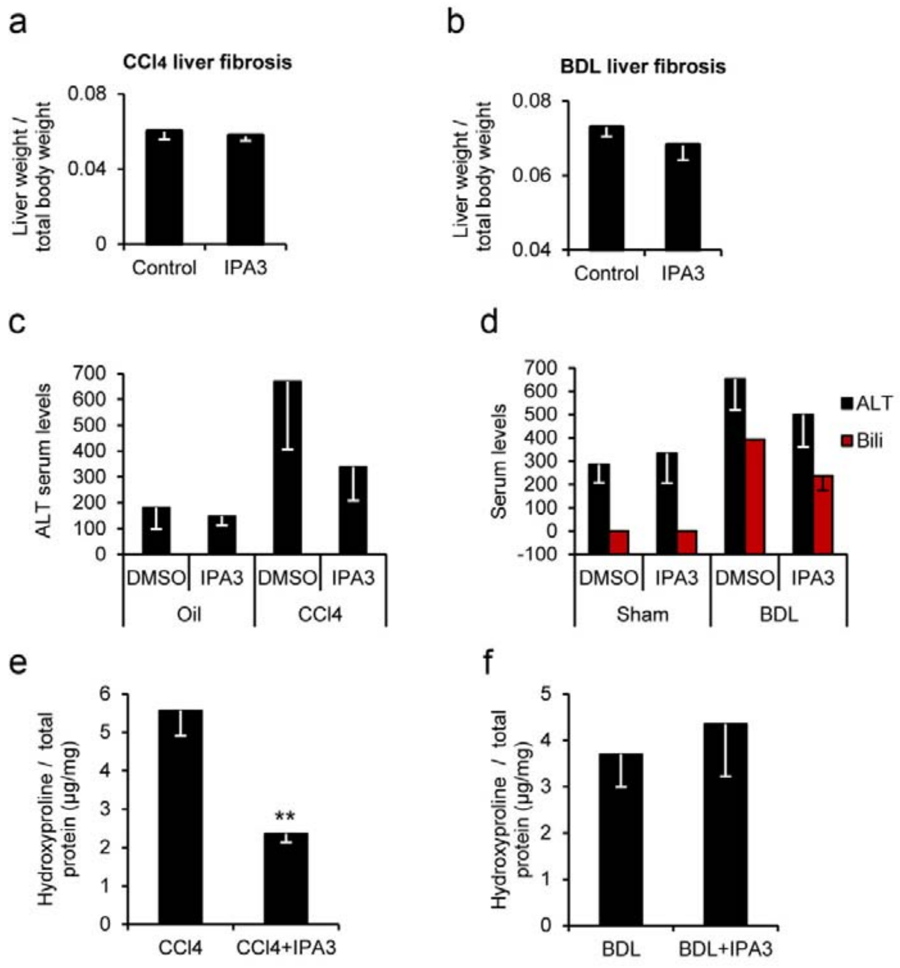

Supplementary Figure 13 Characterisation of livers following IPA3 treatment in CCl_4 and BDL models of liver fibrosis. (**a, b**) IPA3 treatment did not alter the liver weight/body weight ratio compared to the DMSO control group in CCl_4 (**a**) or BDL (**b**) models of liver fibrosis. (**c, d**) Non-significant improvements seemed apparent in liver function following IPA3 treatment compared to DMSO control for serum ALT (CCl_4 model; **c**) and ALT & bilirubin (BDL; **d**). (**e, f**) Hydroxyproline levels were lowered in IPA3-treated CCl_4 liver fibrosis (a model of parenchymal liver disease) but not in the BDL model (a more restricted peribiliary model of fibrosis). Two-tailed unpaired *t*-test was used for statistical analysis. Data are shown as means ± s.e.m. $^{**}P<0.01$.

Supplementary Figure 14. Raw immunoblotting data files

Fig 1. c,d

ITGB1 on Ethanol (E) and Tamoxifen (T) treated activated $Itgb1^{fl/fl}$BactinCreER+ mHSCs

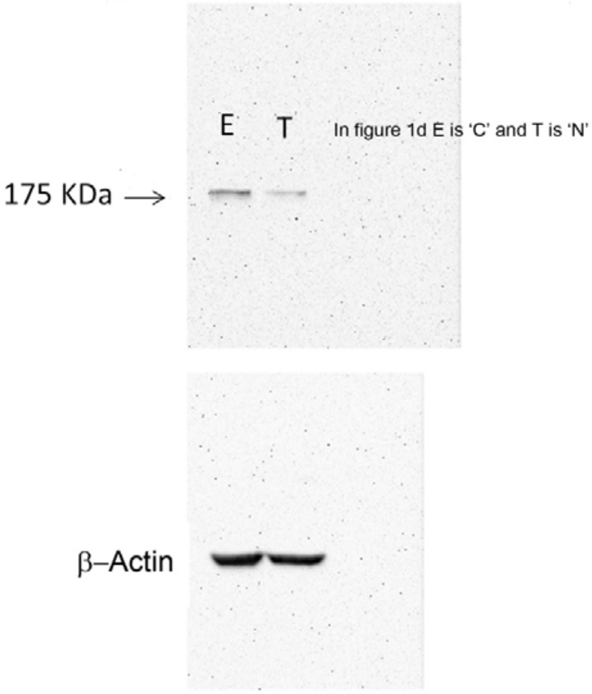

Fig 1. c,d

SOX9 (62KDa) and αSMA (42kDa) on Quiescent (Q), Ethanol (E) and Tamoxifen (T) treated activated $Itgb1^{fl/fl}$BactinCreER+ mHSCs (n= 3)

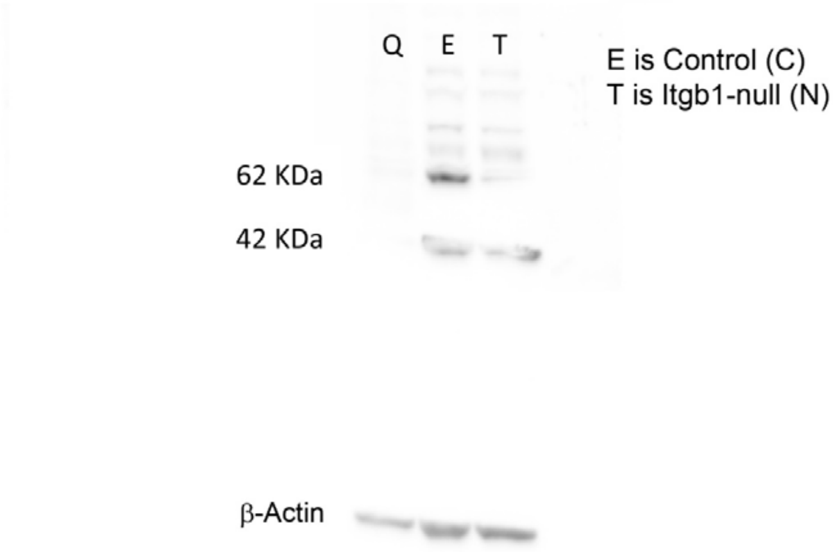

Figure 3a and b

MYL9 on Quiescent (Q) and Activated (A) rHSCs (n= 3)

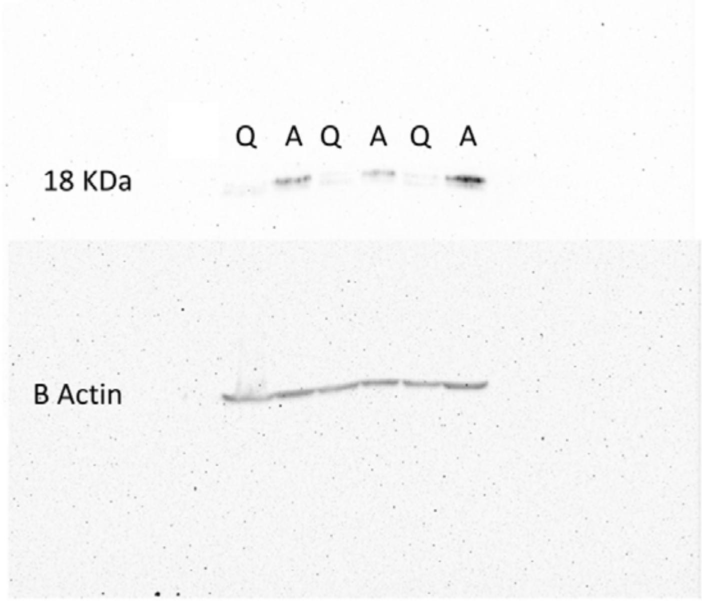

Figure 3a and b

YAP on Quiescent (Q) and Activated (A) rHSCs (n= 3)

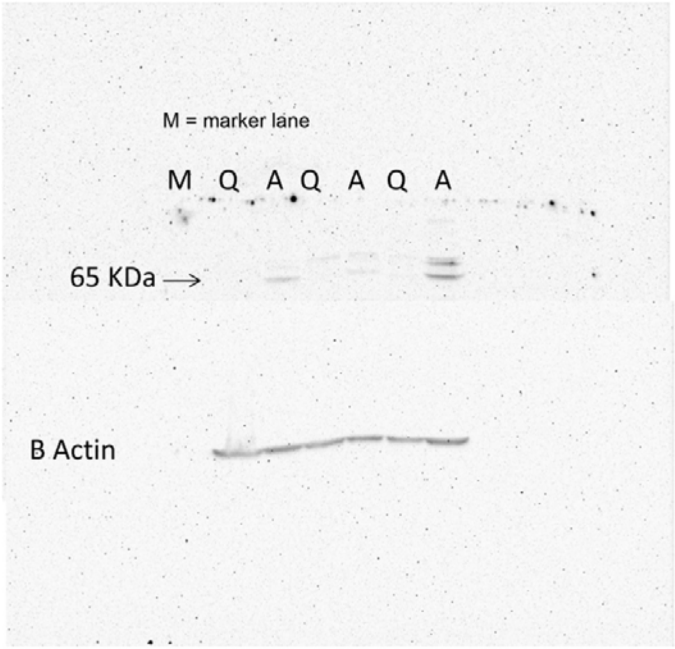

Figure 3c and d

MLY9 on Quiescent (Q), Ethanol (E) and Tamoxifen (T) treated activated Itgb1$^{fl/fl}$BactinCreER+ mHSCs (n= 3)

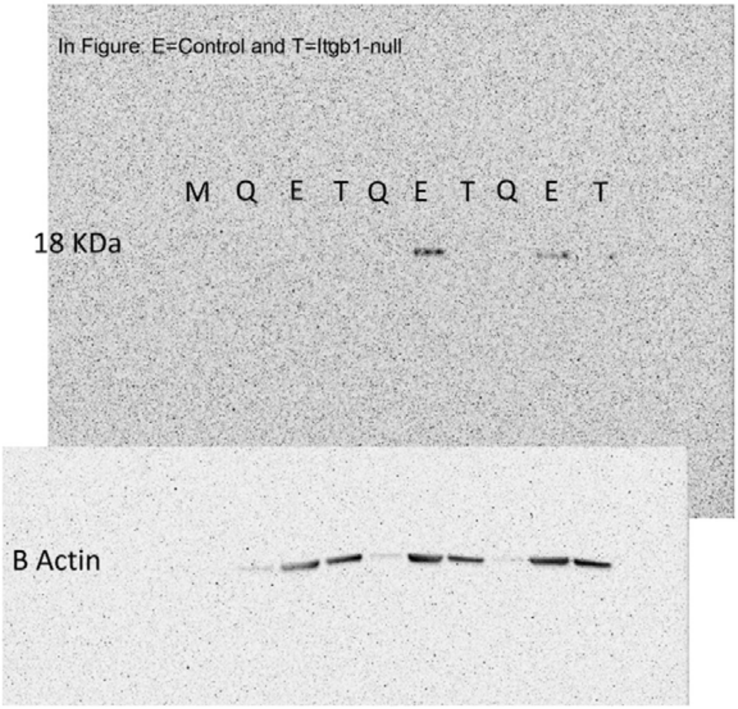

Figure 3c, d and h

YAP on Quiescent (Q), Ethanol (E) and Tamoxifen (T) treated activated Itgb1$^{fl/fl}$BactinCreER+ mHSCs (n= 3)

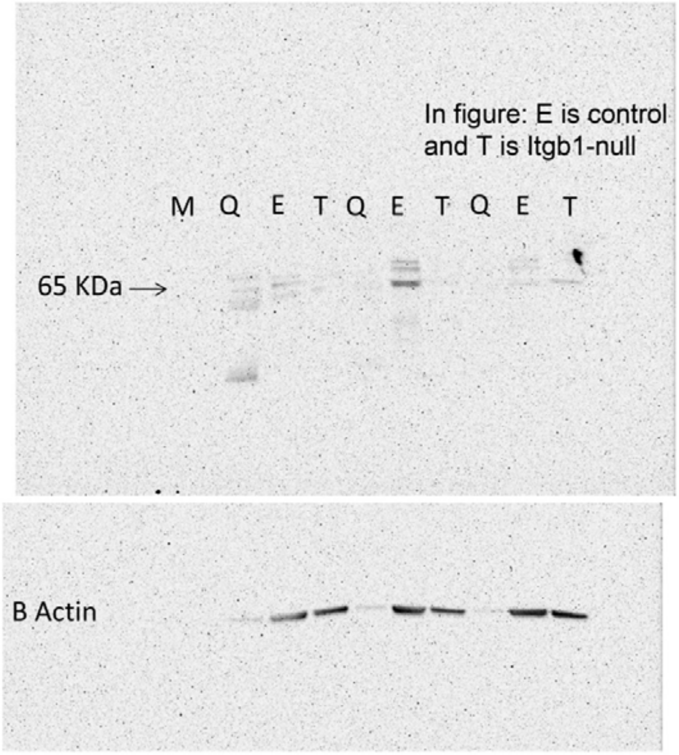

Figure 3h

phospoYAP on Quiescent (Q), Ethanol (E) and Tamoxifen (T) treated activated Itgb1$^{fl/fl}$BactinCreER+ mHSCs (n= 3)

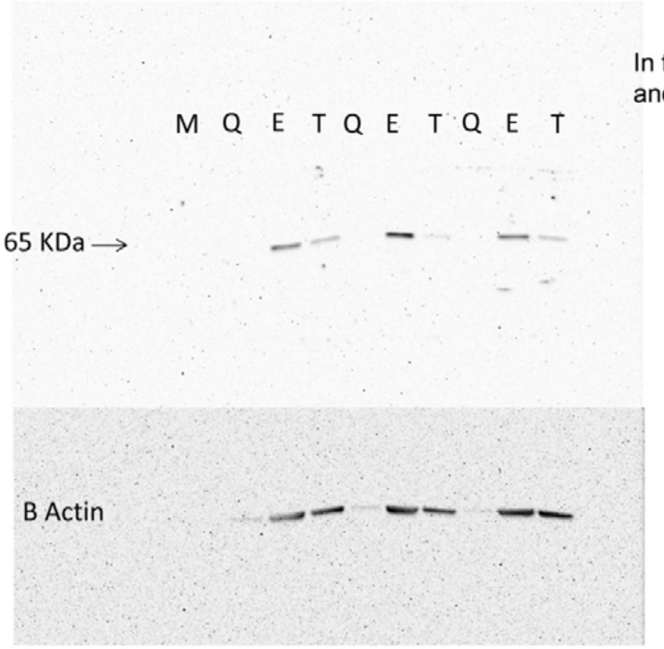

In figure: E is control and T is Itgb1-null

Figures 3g, 5c and Supplementary Figures 2a and 8: ITGA11 siRNA1

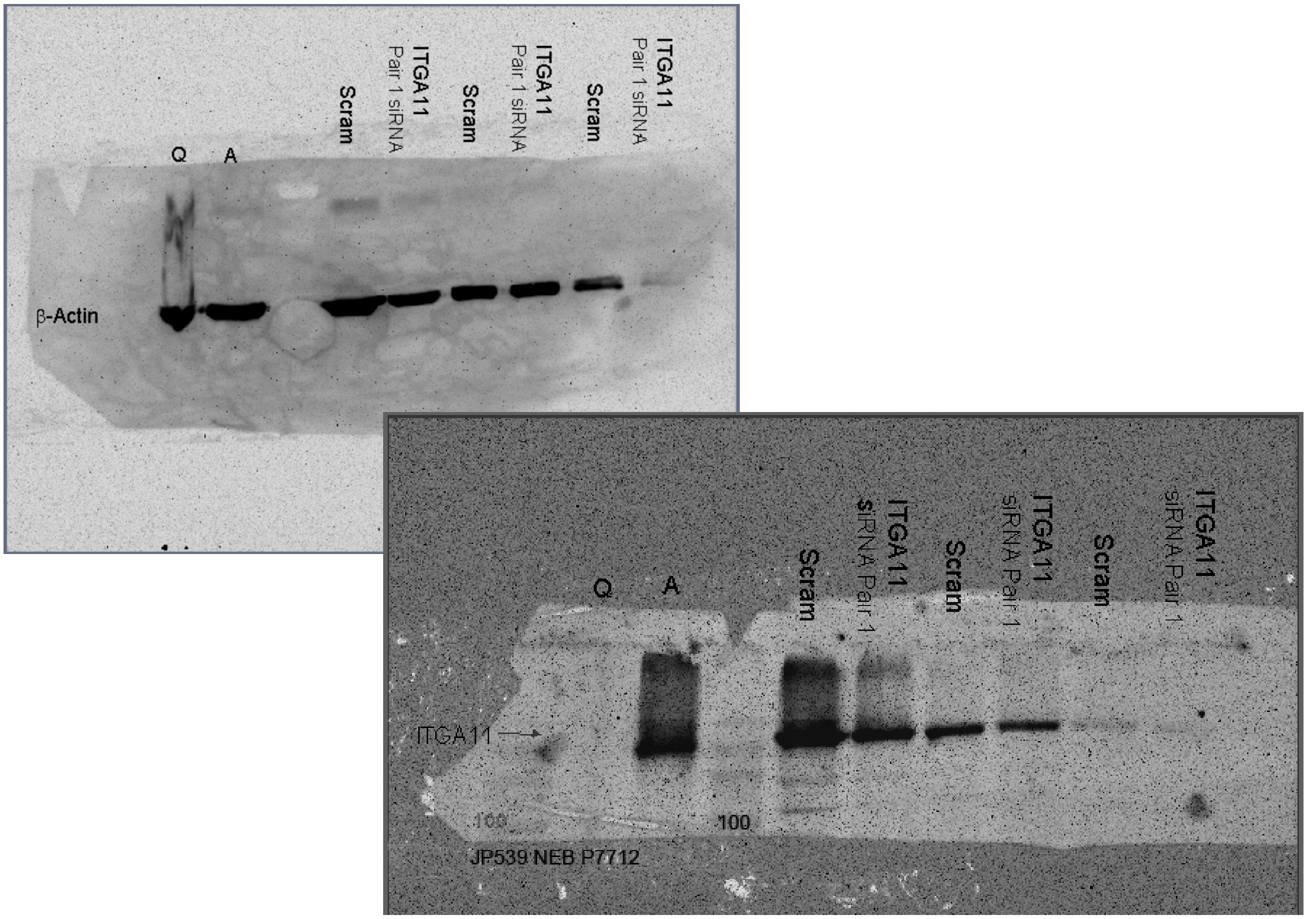

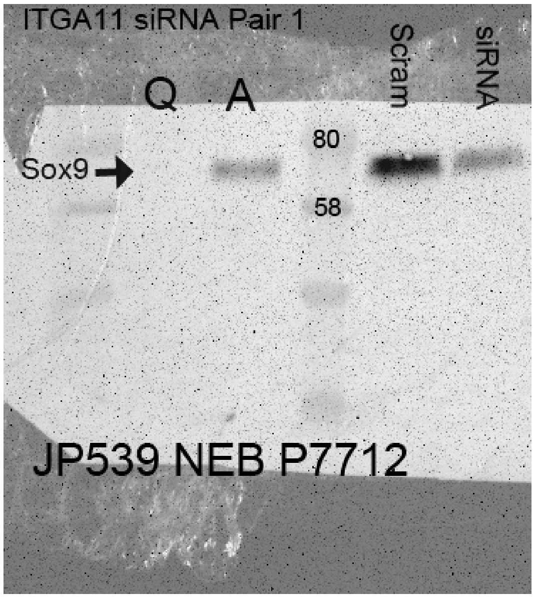
ITGA11 siRNA Pair 1
Q
A
Scram
siRNA
80
Sox9
58
JP539 NEB P7712

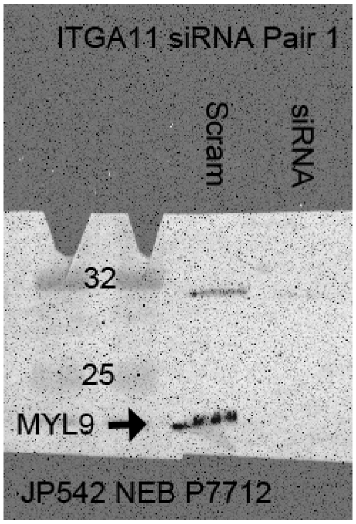
ITGA11 siRNA Pair 1
Scram
siRNA
32
25
MYL9
JP542 NEB P7712

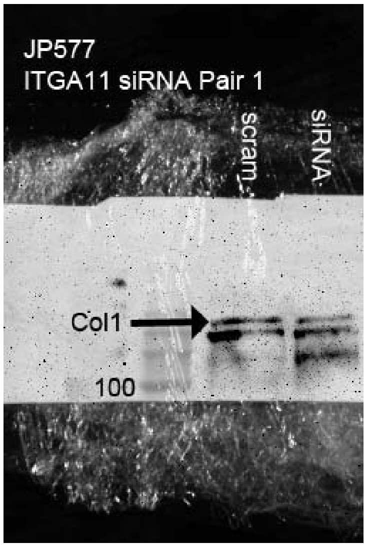
JP577
ITGA11 siRNA Pair 1
scram
siRNA
Col1
100

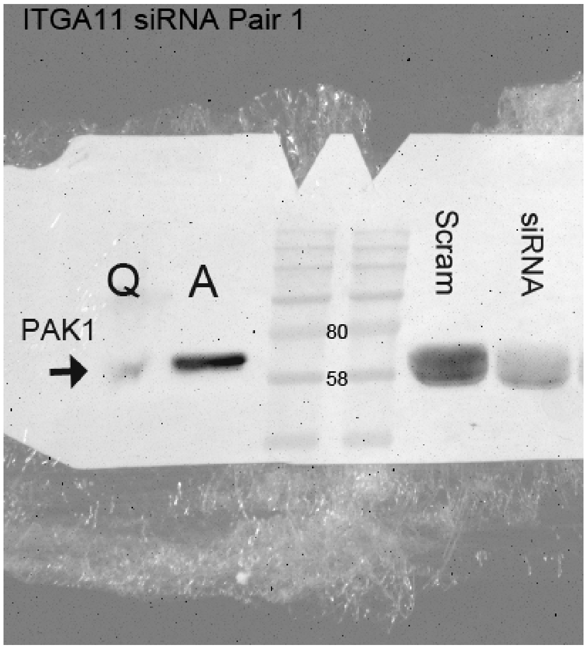
ITGA11 siRNA Pair 1
Q
A
Scram
siRNA
PAK1
80
58

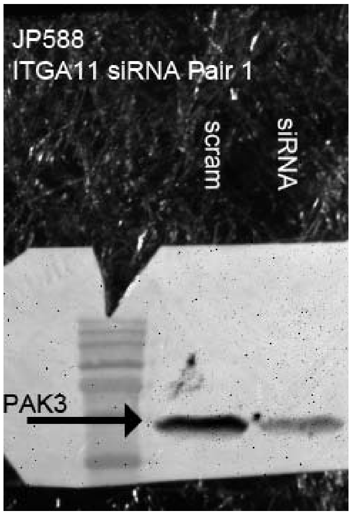
JP588
ITGA11 siRNA Pair 1
scram
siRNA
PAK3

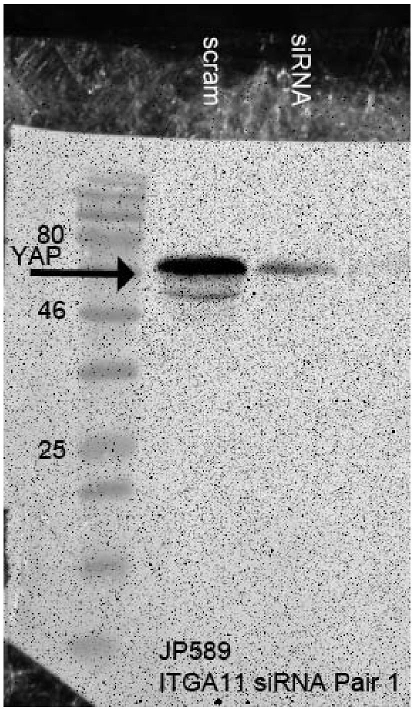

Figures 3g, 5c and Supplementary Figures 2a and 8: ITGA11 siRNA2

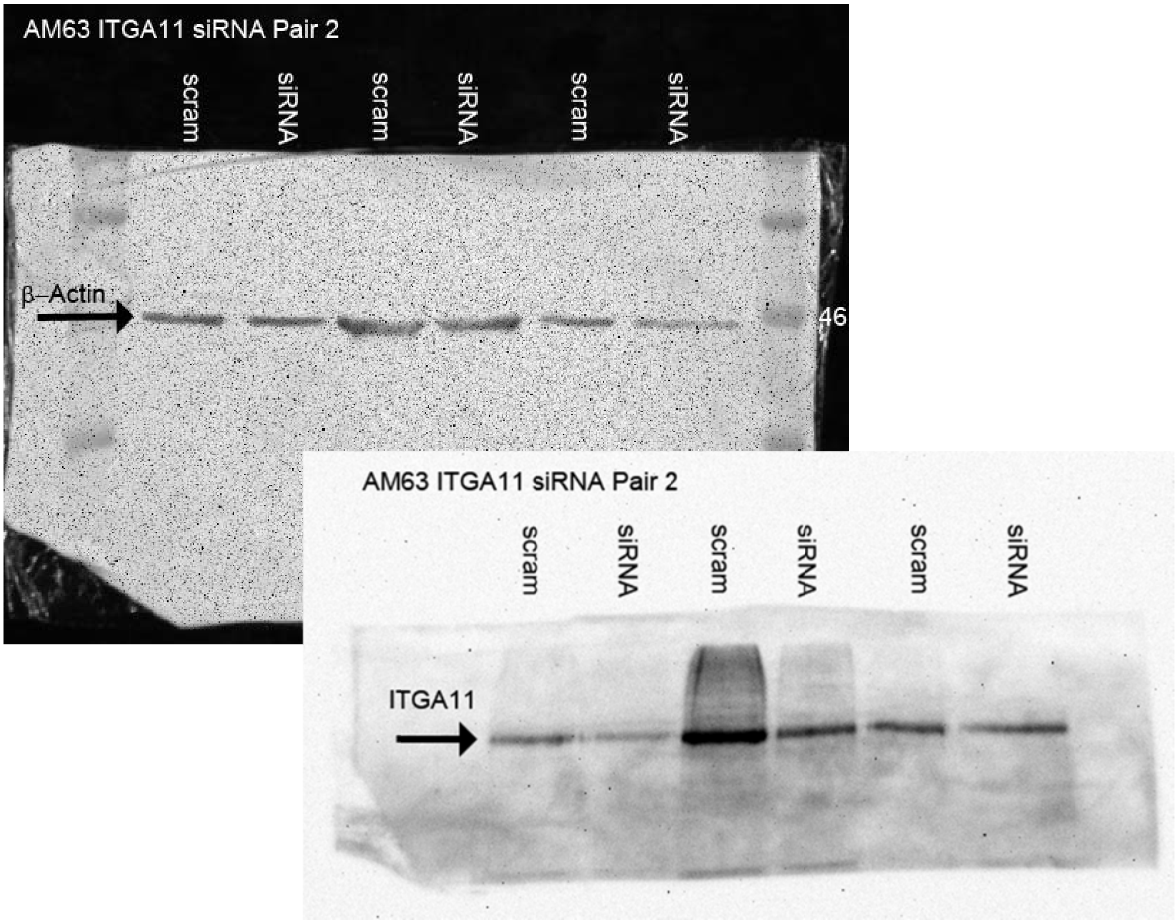

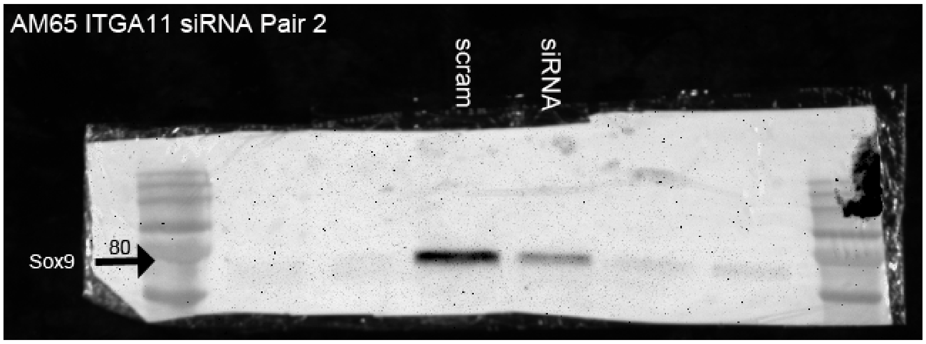
AM65 ITGA11 siRNA Pair 2
scram
siRNA
80
Sox9

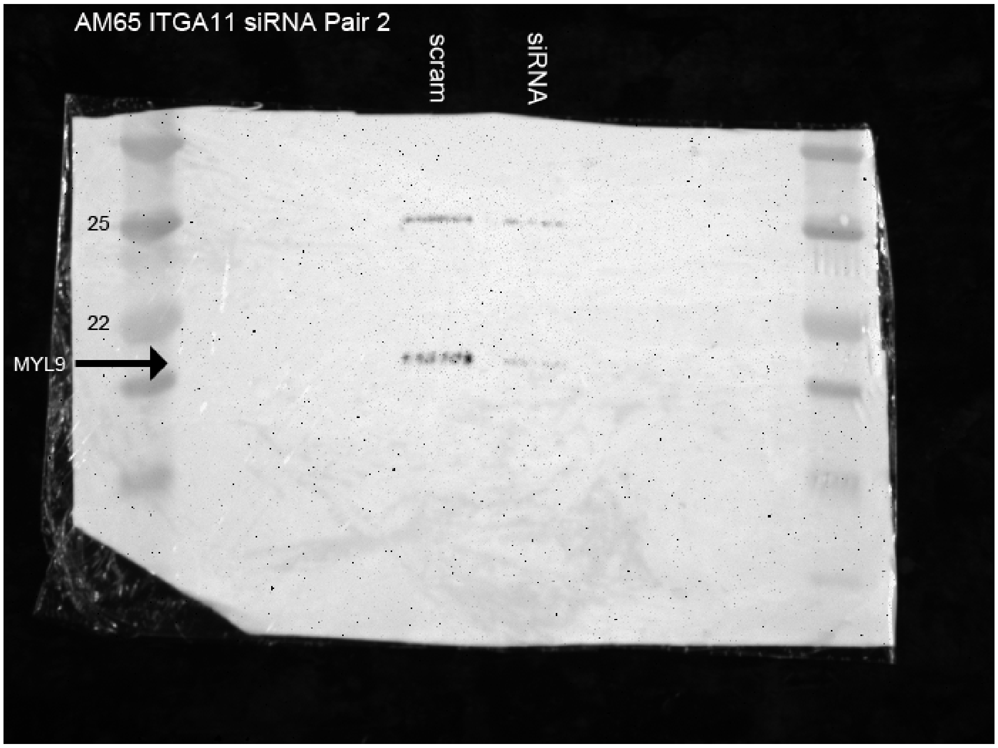
AM65 ITGA11 siRNA Pair 2
scram
siRNA
25
22
MYL9

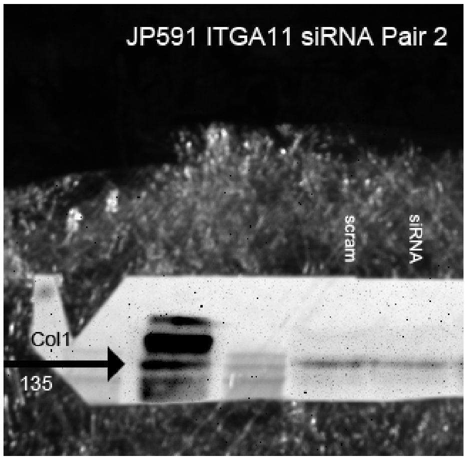
JP591 ITGA11 siRNA Pair 2
scram
siRNA
Col1
135

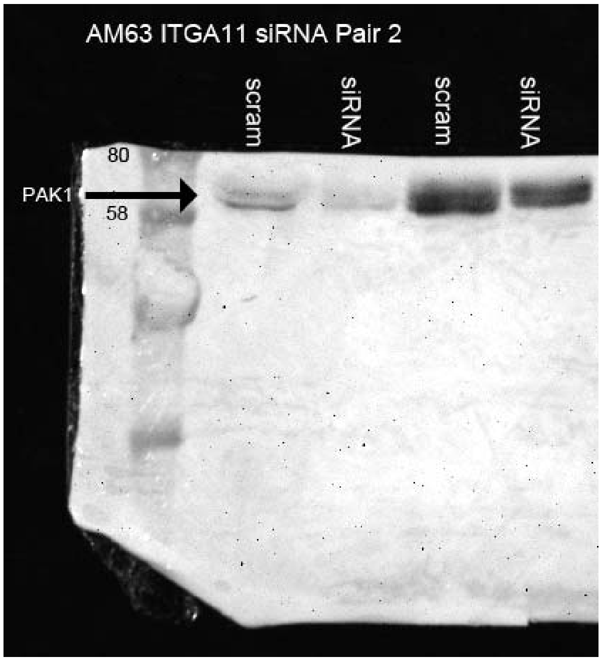
AM63 ITGA11 siRNA Pair 2
scram
siRNA
scram
siRNA
80
PAK1
58

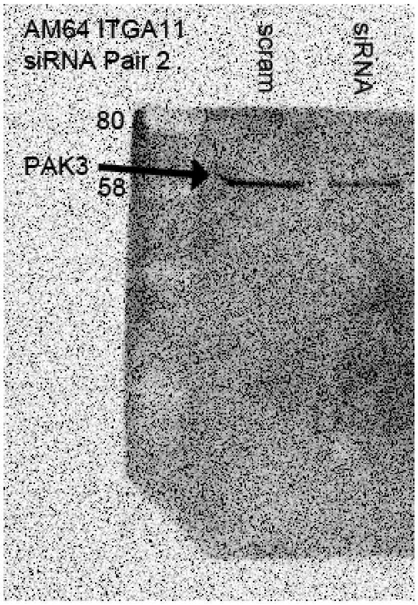
AM64 ITGA11
siRNA Pair 2
scram
siRNA
80
PAK3
58

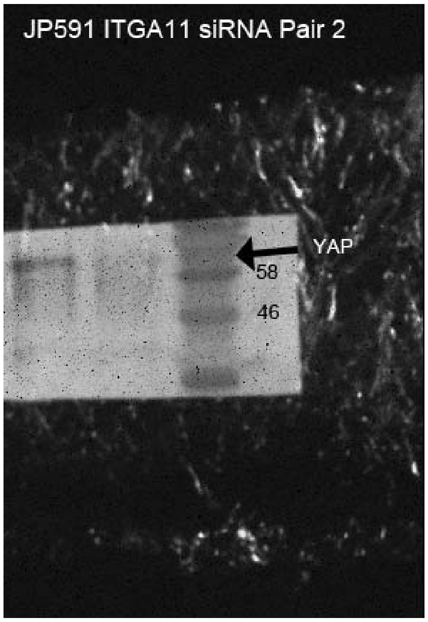
JP591 ITGA11 siRNA Pair 2
YAP
58
46

Figure 5a

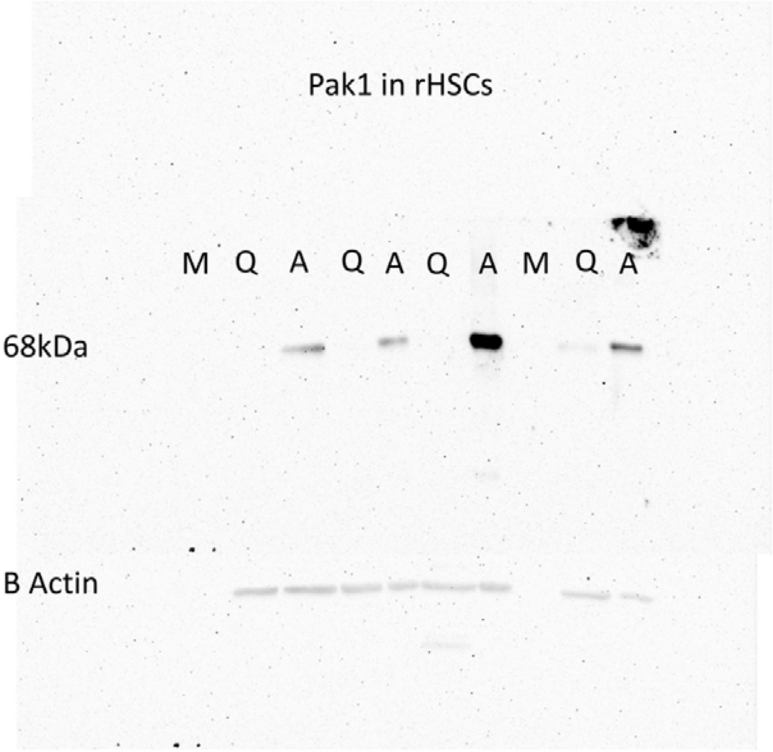

Figure 5a

PAK2 in rHSCs

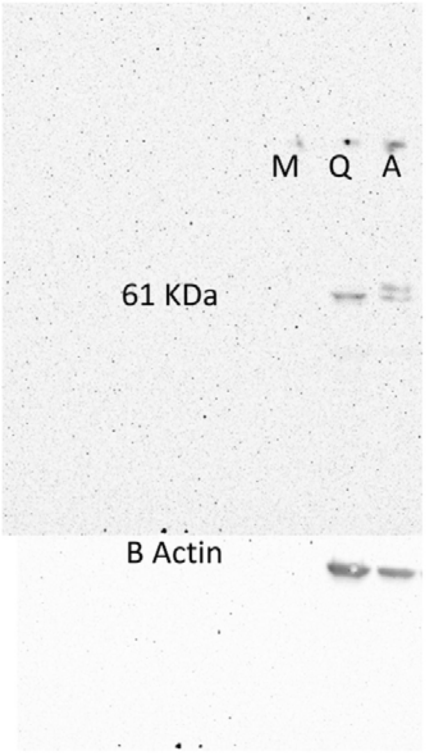

Figure 5a

PAK3 in HSCs

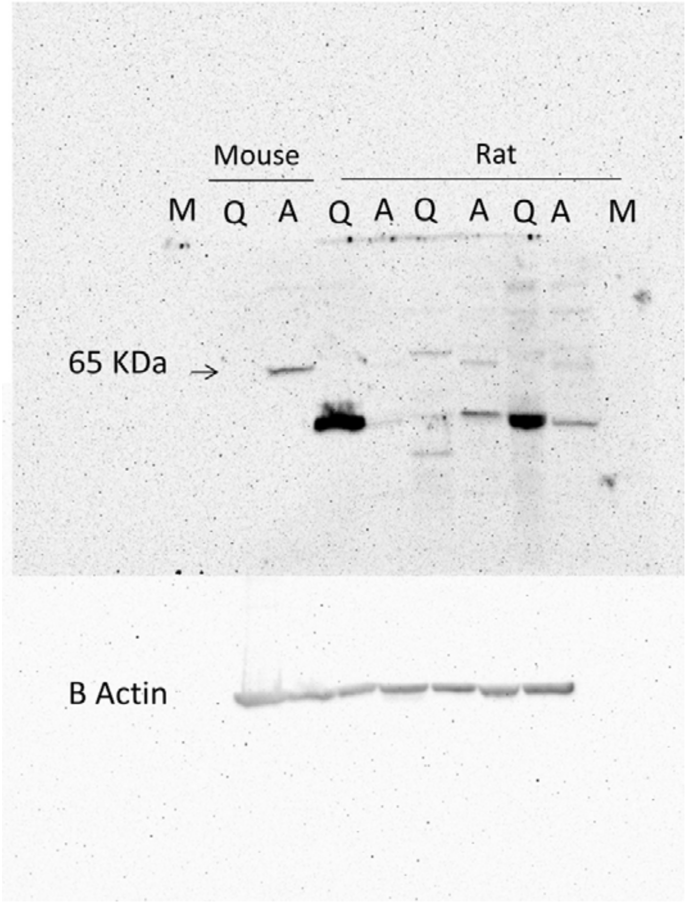

Figure 5b:

Q: Quiescent HSCs

A: Activated HSCs + vehicle = Control

Tx: Activated HSCs + Tamoxifen = Itgb1-null

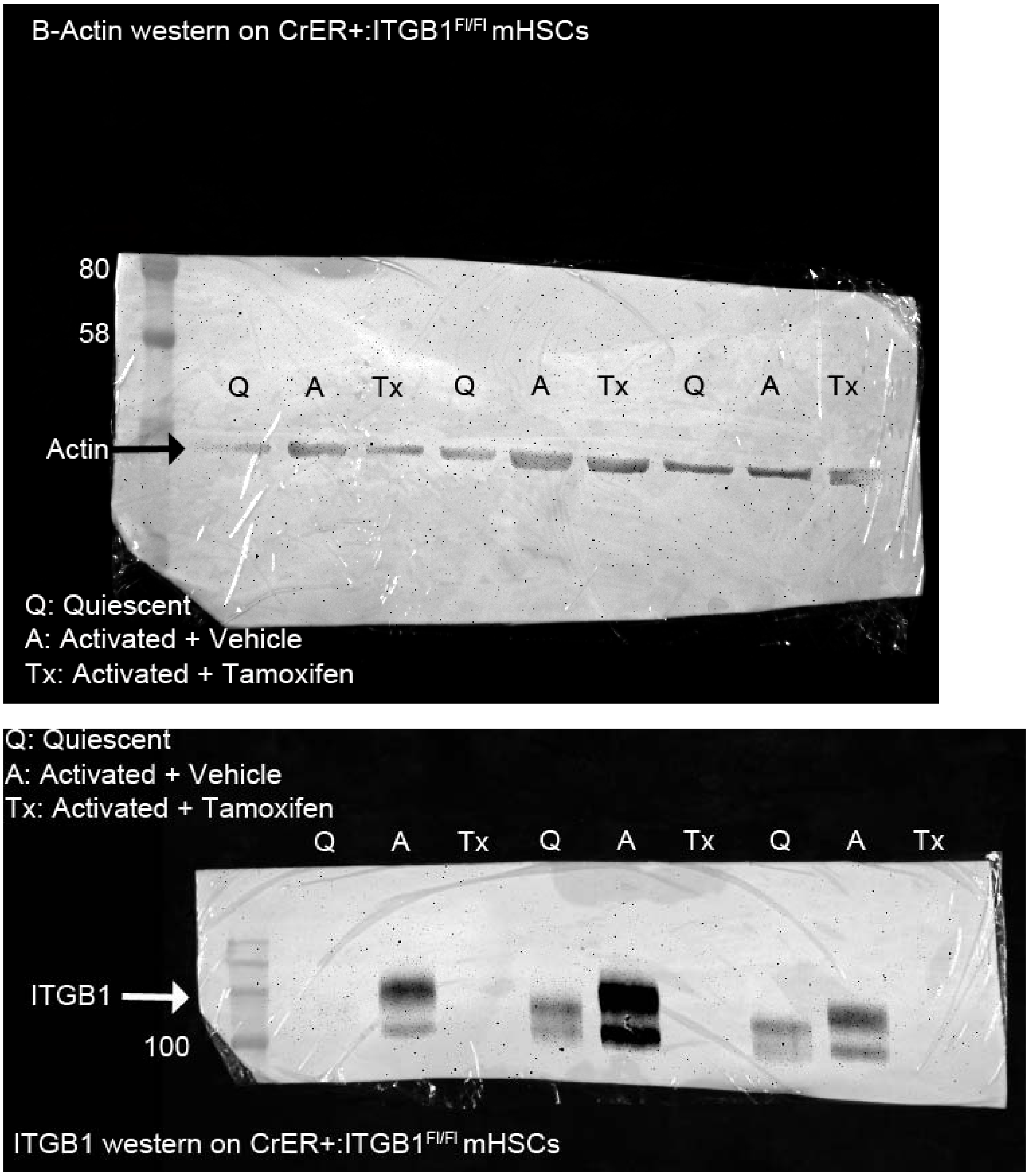

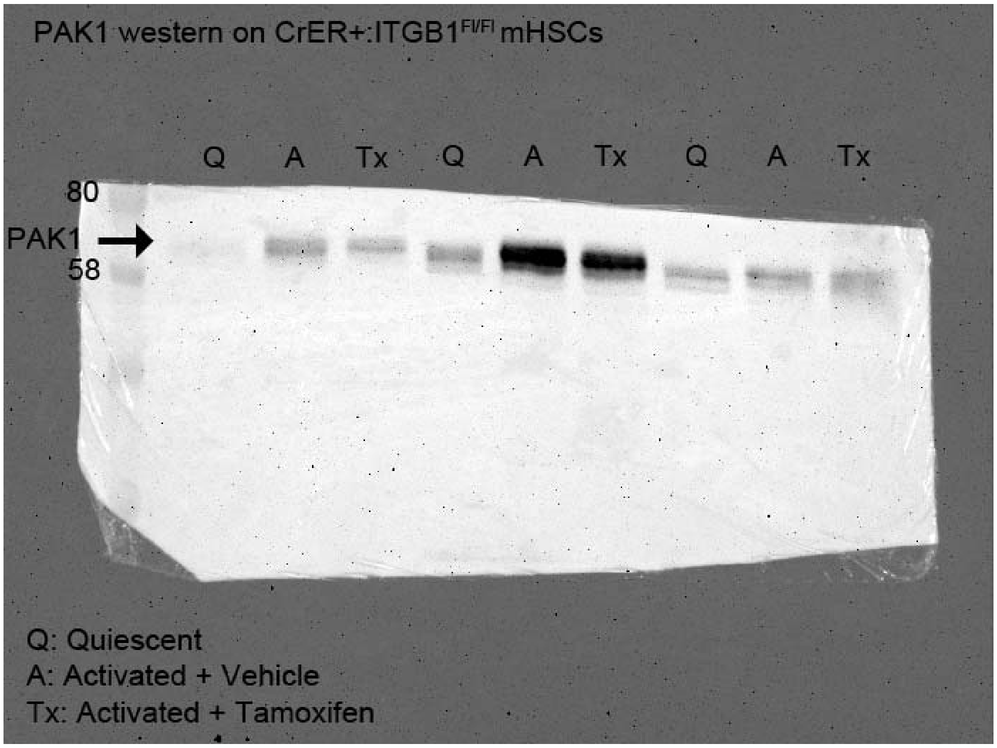
PAK1 western on CrER+:ITGB1$^{Fl/Fl}$ mHSCs
Q A Tx Q A Tx Q A Tx
80
PAK1
58
Q: Quiescent
A: Activated + Vehicle
Tx: Activated + Tamoxifen

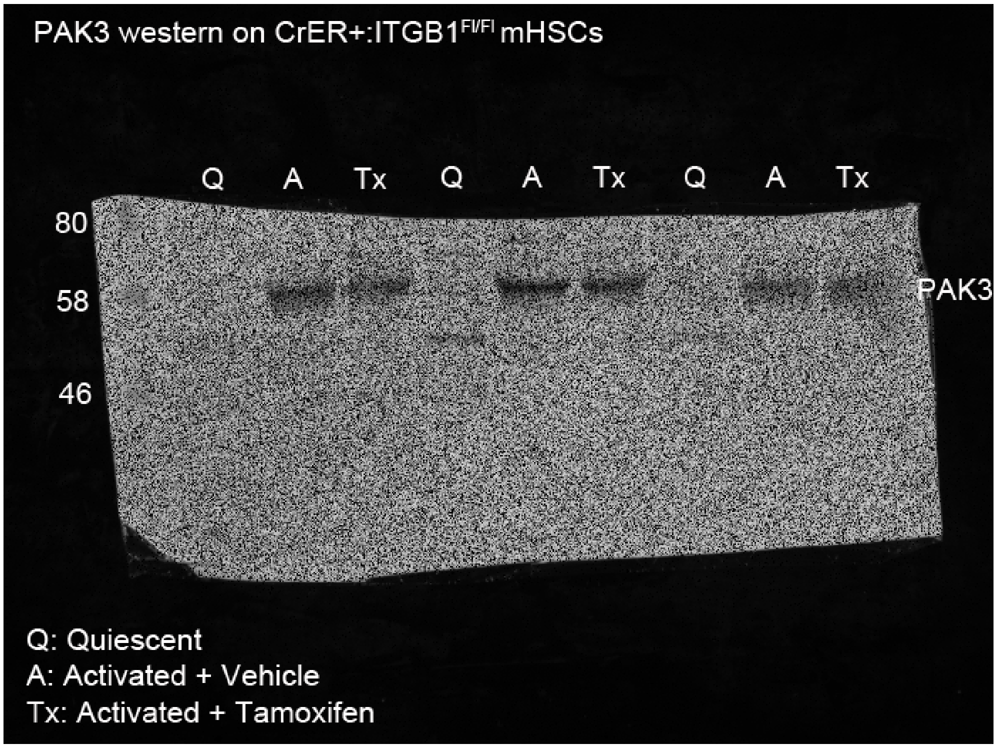
PAK3 western on CrER+:ITGB1$^{Fl/Fl}$ mHSCs
Q A Tx Q A Tx Q A Tx
80
58
46
PAK3
Q: Quiescent
A: Activated + Vehicle
Tx: Activated + Tamoxifen

Figure 5d and e PAK1 siRNA1

Scrambled (S) and siRNA (K) treated mHSCs

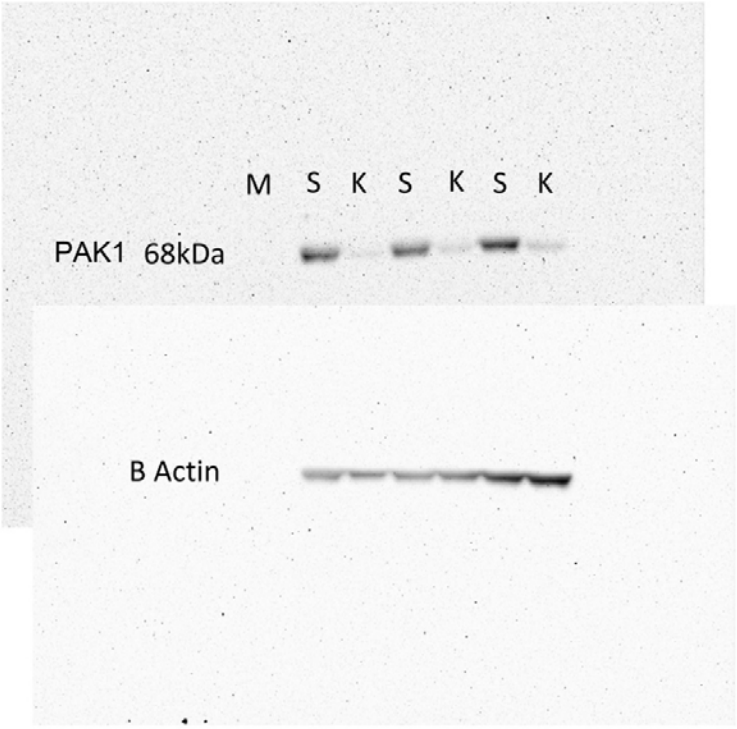

Figure 5d and e PAK1 siRNA1

Scrambled (S) and siRNA (K) treated mHSCs

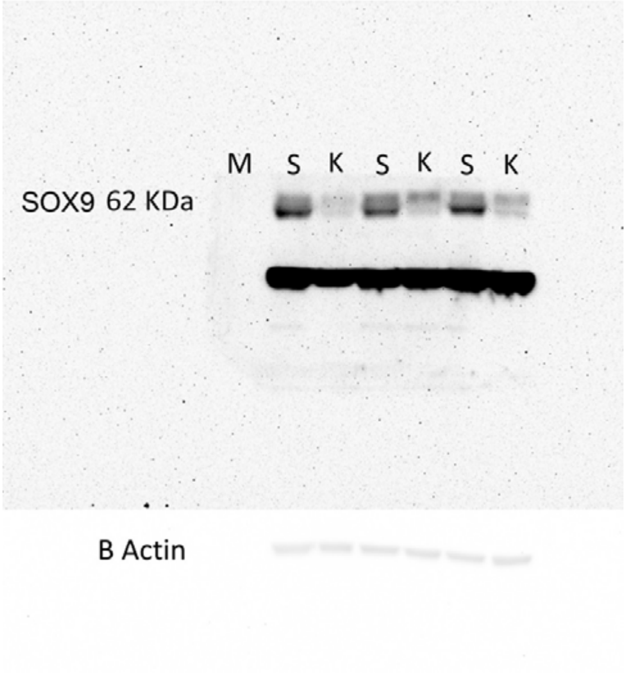

Figure 5d and e PAK1 siRNA1

Scrambled (S) and siRNA (K) treated mHSCs

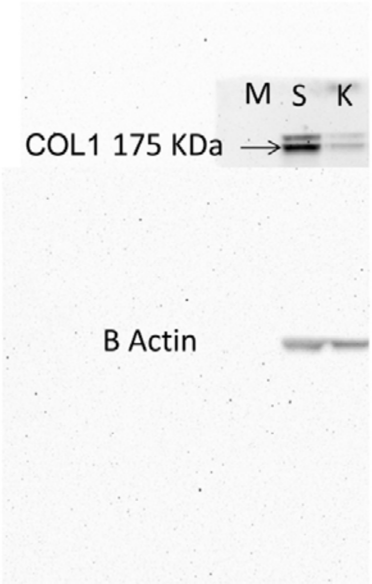

Figure 5d and e PAK1 siRNA2

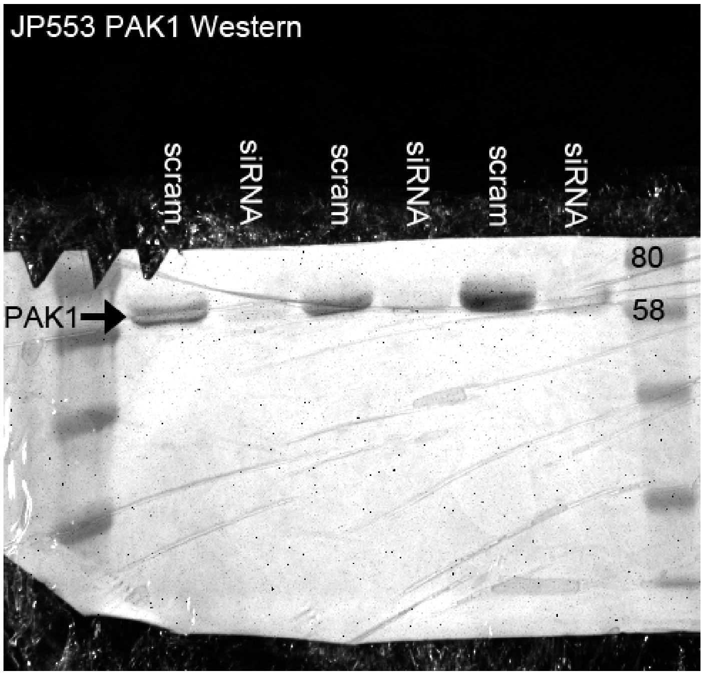

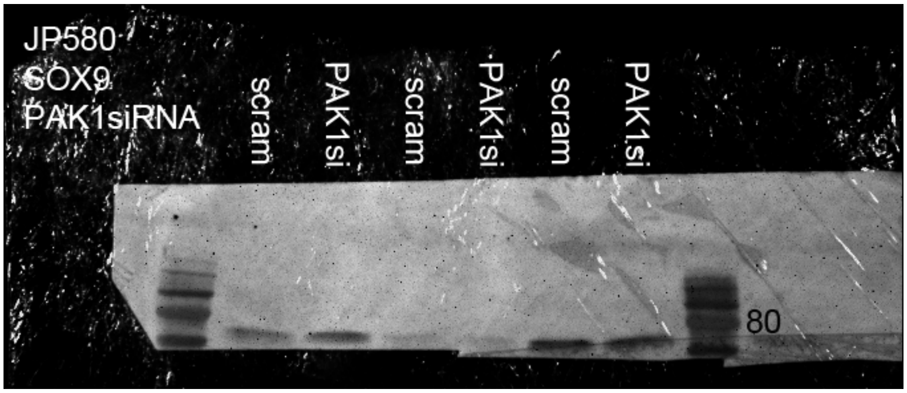

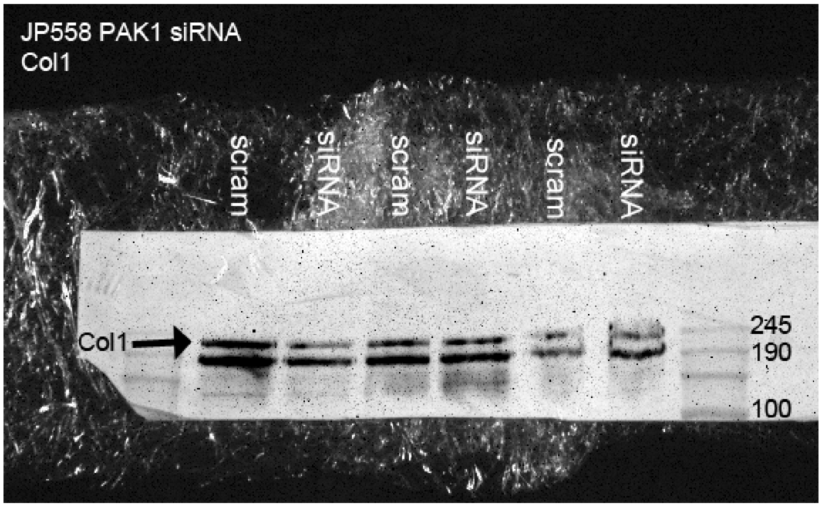

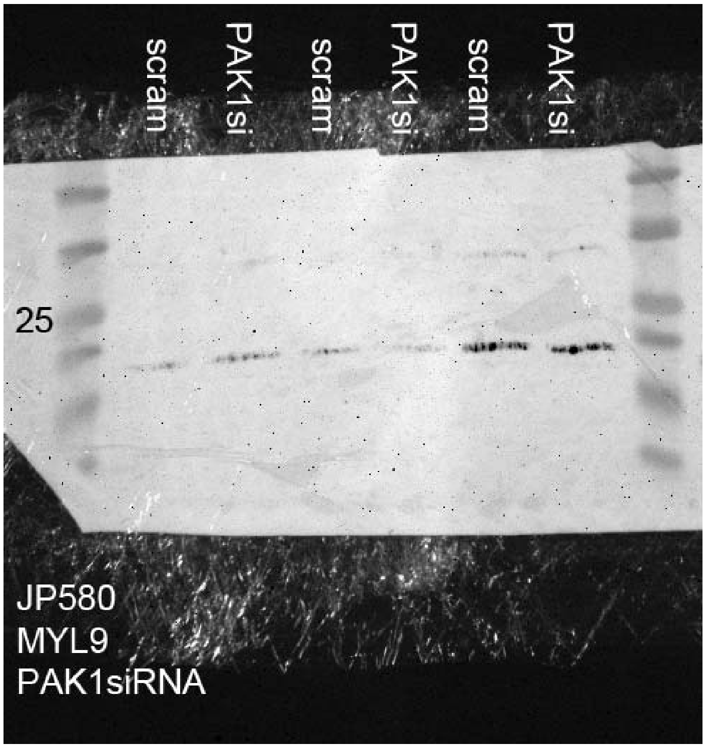

Figure 5f

COL1 (175KDa) and SOX9 (62KDa) on Control(C) and IPA3 treated (T) rHSCs.

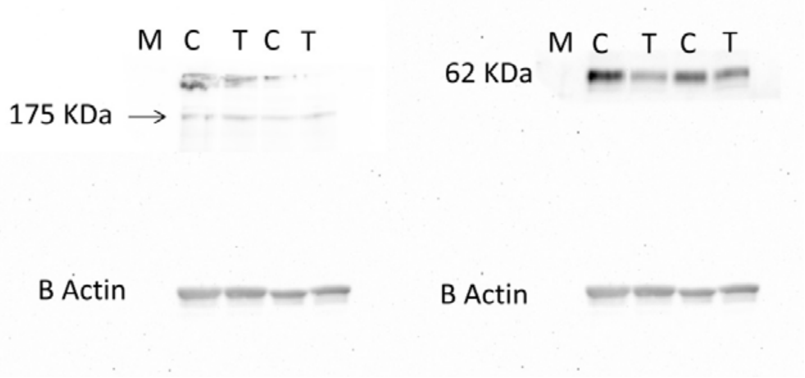

Figure 5f

Activated human HSCs treated with DMSO (C) or IPA3 (T).

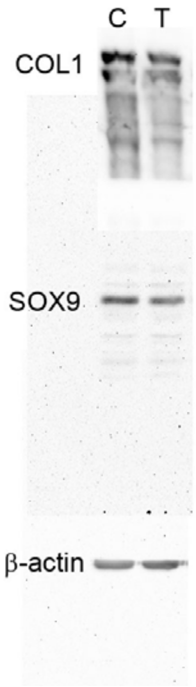

Supplementary Figure 1a, b

ITGB1 on rHSCs timecourse in culture activation days 0 - 14

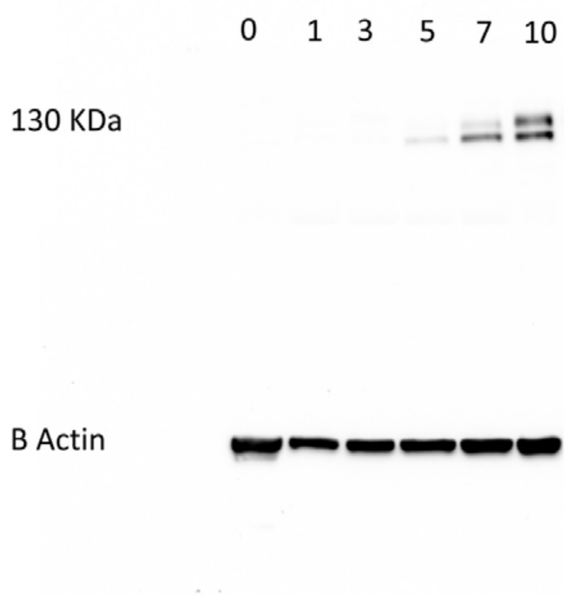

Supplementary Figure 1a, b

COL1 on rHSCs timecourse in culture activation days 0 - 14

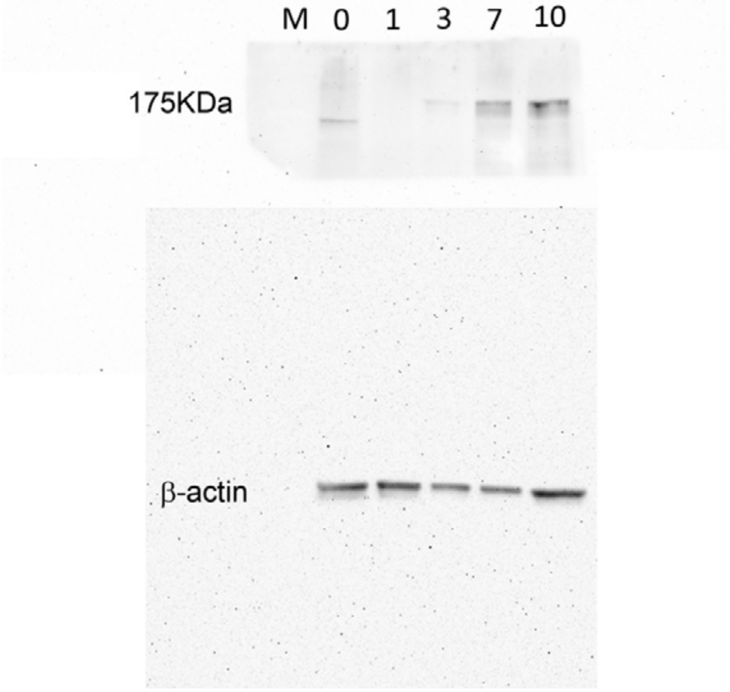

Supplementary Figure 1a, b

αSMA on rHSCs timecourse in culture activation days 0 - 14

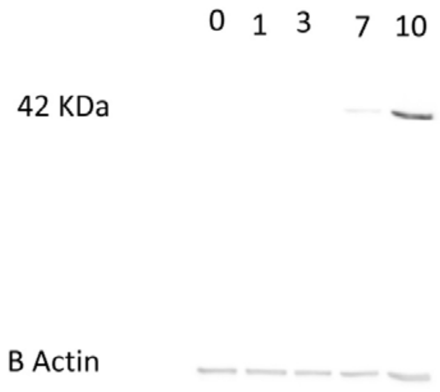

Supplementary Figure 1d

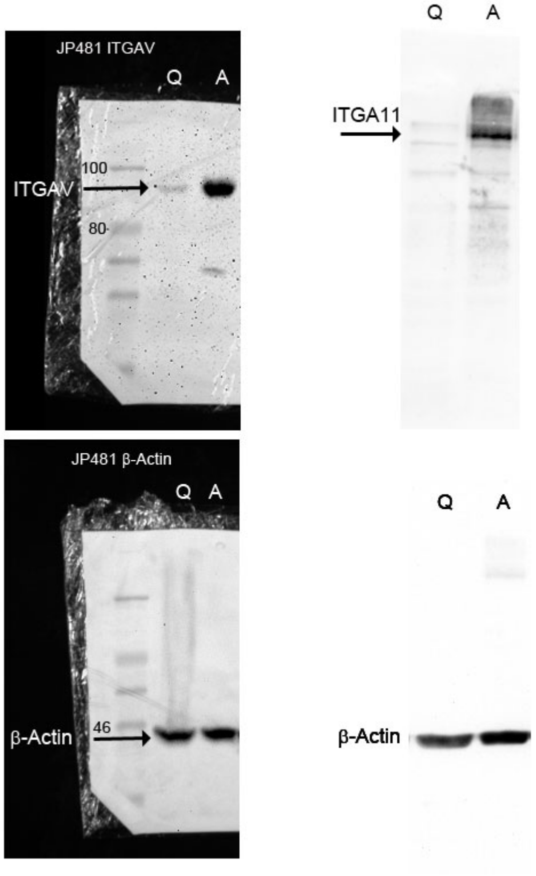

Supplemental Figure 1e

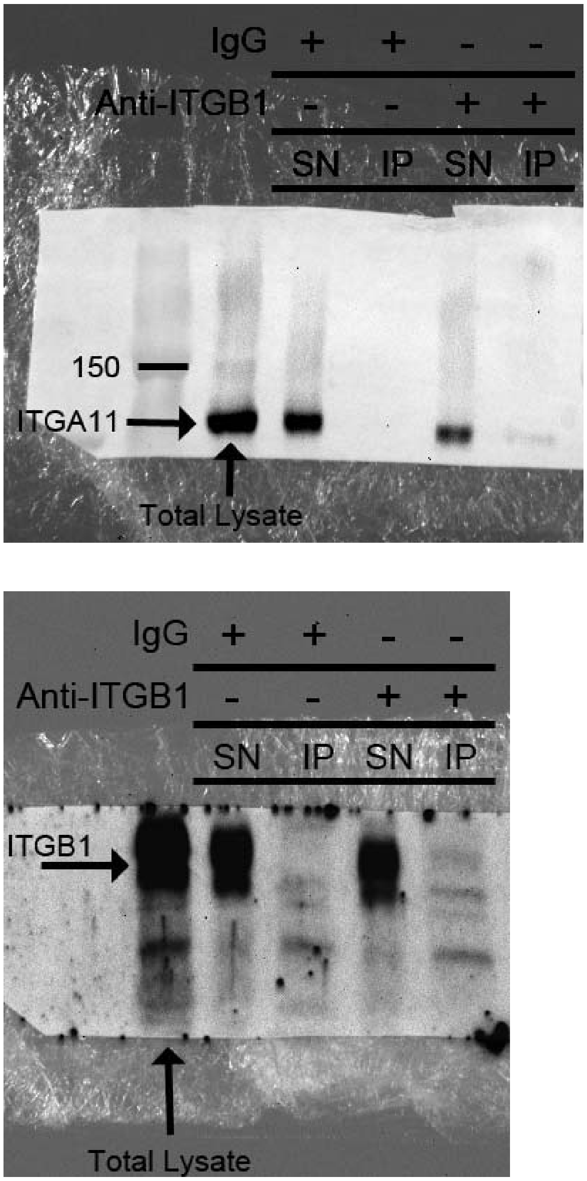

Supplemental Figure 11

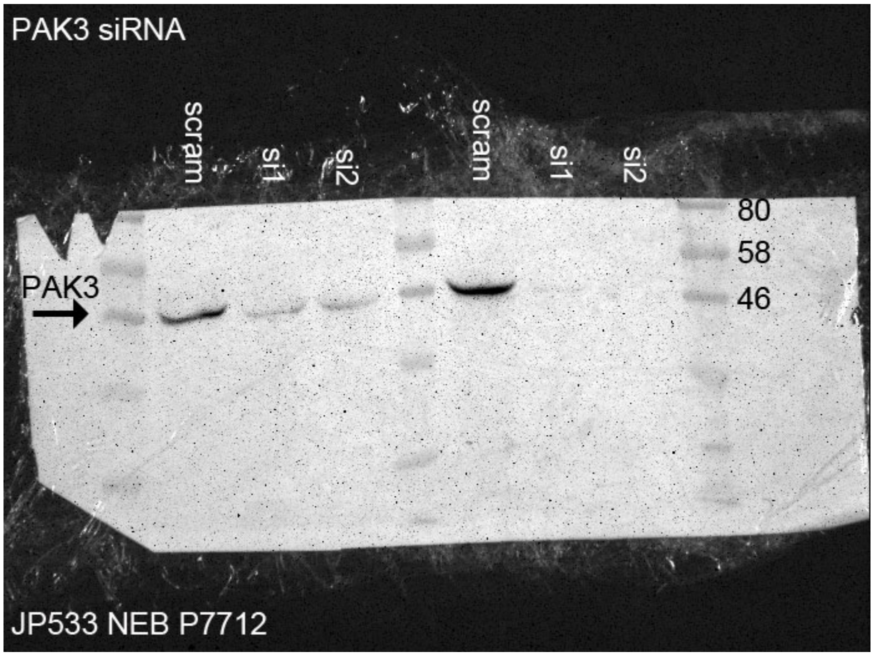

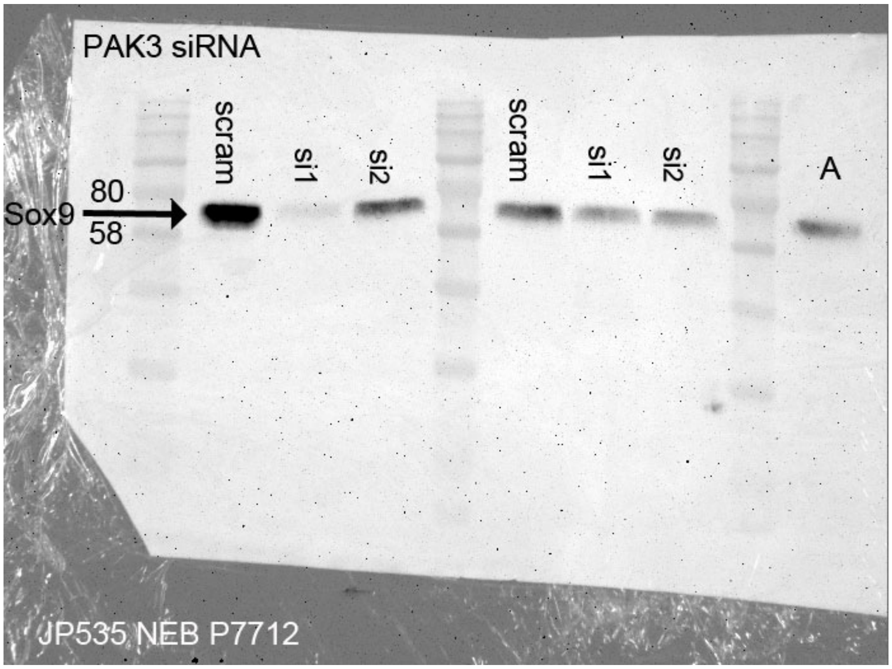

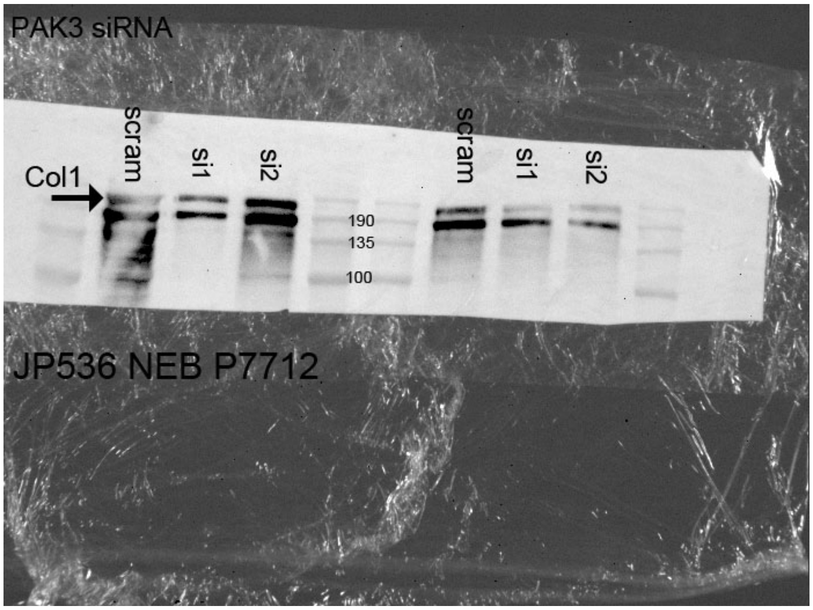
PAK3 siRNA
Col1
scram
si1
si2
190
135
100
scram
si1
si2
JP536 NEB P7712

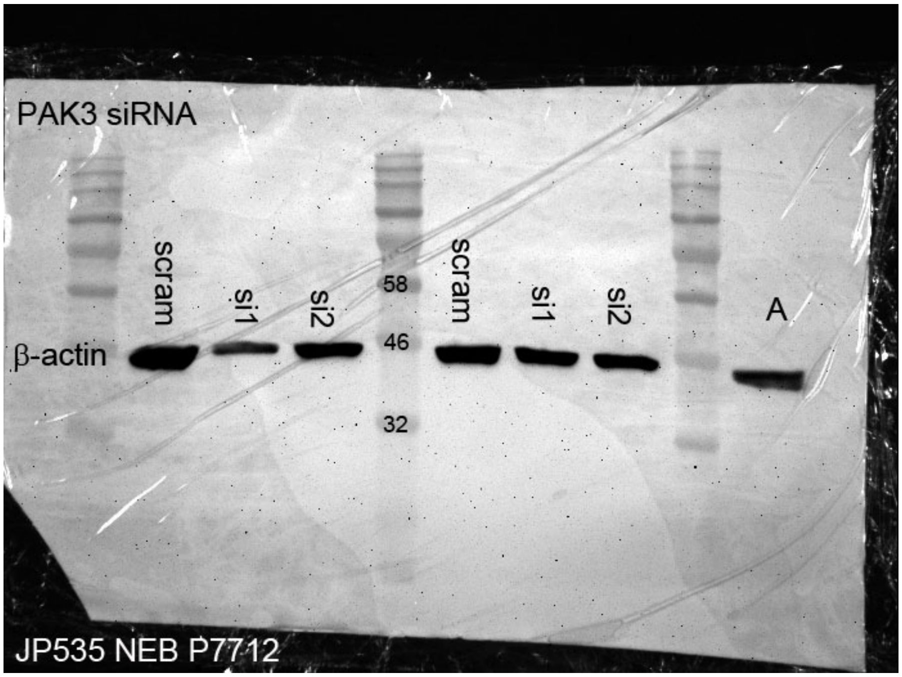
PAK3 siRNA
scram
si1
si2
58
46
32
scram
si1
si2
A
β-actin
JP535 NEB P7712

**siRNA target gene**	**Cat. No.**	**Species**	**Target sequence**
Itga11 (siRNA1)	SI00244566	mouse	CACGCCCTATCTGGACCTATA
	SI02699046	mouse	TACGACCTTTACTGTCAGAAA
Itga11 (siRNA2)	SI02748179	mouse	CCGCCCTGTAGTTCAAATCAA
	SI02722755	mouse	CAGCTTCTACCTGGTGGGAAA
Pak1(siRNA1)	SI01368598	mouse	CTGGGCATTATGGCAATTGAA
Pak1(siRNA2)	S101368591	mouse	CTCGCTTGCTTCAAACATCAA
Pak3 (siRNA2)	SI01368682	mouse	CCCACTGAGGATGAACAGTAA
Pak3 (siRNA1)	SI04418967	mouse	TAGCAGCACATCAGTCGAATA

Supplementary Table 1 siRNA target sequences.

Target	Species	Forward	Reverse	Application
Acta2	Mouse	GTCCCAGACATCAGGGAGTAA	TCGGATACTTCAGCGTCAGGA	qPCR
ActinB	Mouse	GCTGTATTCCCCTCCATCGTG	CACGGTTGGCCTTAGGGTTCAG	qPCR
ActinB	Rat	CCCGCGAGTACAACCTTCT	CGTCATCCATGGCGAACT	qPCR
Bambi	Rat	TGTGCTGCTCACCAAAGGCGA	AGCAGGCACTAAGCTCGGACT	qPCR
ChIP -ve	Mouse	CTGAGGCAGTCGAAGGAGAG	ACACTGGCTGCCAAAATGTA	ChIP
Col1a1	Mouse	TGTTGGCCCATCTGGTAAAGA	CAGGGAATCCGATGTTGCC	qPCR
GusB	Mouse	GCAGTTGTGTGGGTGAATGG	GGGTCAGTGTGTTGTTGATGG	qPCR
GusB	Rat	CTCTGGTGGCCTTACCTGAT	AGGTGTTGTCATCGTCACCTC	qPCR
Hspa1a/b	Rat	TGTCCCTCAAGAGCCCAACCC	TTGGCTCTCCACACAGGAACCC	qPCR
Itga1	Rat	GCAACCGGAAGCGAGAGCTGG	TAGCAGCAGTAGCCCCGCGA	qPCR
Itga10	Rat	GGCCTGTGCCCCTCTCTGGT	GGACAACGTTGGGCGGTCGG	qPCR
Itga11	Mouse	GATACGCTGTGGCCGTTTTG	AGAAGTGCTTGTCGTCAGGG	qPCR
Itga11	Rat	ACCCGCACGGCATTTGGCAT	TCGTGGGATTCCCCGTCCGT	qPCR
Itga2	Rat	TGTGCGCACCCCCAAAAGCA	CCGTCAATCTTGAGTGAGCAGTAGC	qPCR
Itga3	Rat	AGTCCTGGTCTGTGCCCATCGG	TCGTTGCCACGCACGTAGCA	qPCR
Itga4	Rat	GGGTACCAACCGGGCACTCC	AATGAGCCAGCGCTTCGTCCC	qPCR
Itga5	Rat	TTTGGCAGTGCAGCAGGGCA	CCACGCGGCCAGTCTTGGTG	qPCR
Itga6	Rat	TGCGGGCACTCAGGTTCGAG	AGGATGATCCACCACGCTATCCCT	qPCR
Itga7	Rat	TCTGGGGAGCGCCTGACCTC	AGGTCTGCCCAGCCATCACTGT	qPCR
Itga8	Rat	GCGCGCACAGCCAGTGTCTT	CCTCTCTGCAGGCCAGGGACA	qPCR
Itga9	Rat	AGACGACGCCTACGACGCCAA	ATGCCCATCTCCTCCTTCTGCCAC	qPCR
Itgb1	Mouse	GCCAAGTGGGACACGGGTGAA	AGCTTGGTGTTGCAAAATCCGCCT	qPCR
Myl9	Mouse	CTCTGCAGCAGGGAAACCC	CTTCTTGGTGGTCTTGGCCT	qPCR
Myl9	Mouse	GACCCTACAAGGAGGGACCA	CTTCCTTCCTCAGGCCAGC	ChIP
Pak1	Mouse	TTAGCCGAATCCAGCCTGTC	CAGCAGCTACTGGCGGTG	qPCR
Pdgfrb	Mouse	TCCAGGAGTGATACCAGCTTT	CAGGAGCCATAACACGGACA	qPCR
Pparg	Rat	TCTCAGTGGAGACCGCCCAGG	GGGAGGACTCCGGGTGGTTCAG	qPCR
Yap1	Mouse	ATTTCGGCAGGCAATACGGA	CATCCTGCTCCAGTGTAGGC	qPCR

Supplementary Table 2 PCR primer sequences and applications.
